# The causality of borrowing: Lexical loans in Eurasian languages

**DOI:** 10.1371/journal.pone.0223588

**Published:** 2019-10-30

**Authors:** Gerd Carling, Sandra Cronhamn, Robert Farren, Elnur Aliyev, Johan Frid

**Affiliations:** 1 Centre for Languages and Literature, Lund University, Lund, Sweden; 2 Institute of Caucasus Studies, Ivane Javakhishvili Tbilisi State University, Tbilisi, Georgia; 3 Humanities Lab, Lund University, Lund, Sweden; Leiden University, NETHERLANDS

## Abstract

All languages borrow words from other languages. Some languages are more prone to borrowing, while others borrow less, and different domains of the vocabulary are unequally susceptible to borrowing. Languages typically borrow words when a new concept is introduced, but languages may also borrow a new word for an already existing concept. Linguists describe two causalities for borrowing: *need*, i.e., the internal pressure of borrowing a new term for a concept in the language, and *prestige*, i.e., the external pressure of borrowing a term from a more prestigious language. We investigate lexical loans in a dataset of 104 concepts in 115 Eurasian languages from 7 families occupying a coherent contact area of the Eurasian landmass, of which Indo-European languages from various periods constitute a majority. We use a cognacy-coded dataset, which identifies loan events including a source and a target language. To avoid loans for newly introduced concepts in languages, we use a list of lexical concepts that have been in use at least since the Chalcolithic (4000–3000 BCE). We observe that the rates of borrowing are highly variable among concepts, lexical domains, languages, language families, and time periods. We compare our results to those of a global sample and observe that our rates are generally lower, but that the rates between the samples are significantly correlated. To test the causality of borrowing, we use two different ranks. Firstly, to test need, we use a cultural ranking of concepts by their mobility (of nature items) or their labour intensity and “distance-from-hearth” (of culture items). Secondly, to test prestige, we use a power ranking of languages by their socio-cultural status. We conclude that the borrowability of concepts increases with increasing mobility (nature), and with increased labour intensity and “distance-from-hearth” (culture). We also conclude that language prestige is not correlated with borrowability in general (all languages borrow, independently of prestige), but prestige predicts the directionality of borrowing, from a more prestigious language to a less prestigious one. The process is not constant over time, with a larger inequality during the ancient and modern periods, but this result may depend on the status of the data (non-prestigious languages often remain unattested). In conclusion, we observe that need and prestige compete as causes of lexical borrowing.

## Introduction

### Background: Lexical borrowing

Lexical borrowing is a topic of major interest in several fields of linguistics, including language contact, historical linguistics, and language typology. There is a rich literature in the area of lexical borrowing, as well as in the adjacent domain of substrate interference. These two phenomena are connected but not necessarily accompanying each other [1]. However, the presence or impact of substrate interference is more problematic to identify, in particular if we move back into history and prehistory [1, 2]. Loans between languages, on the other hand, can normally be secured by chronology of sound changes, also in history and prehistory. The domain of *content words* (e.g., nouns, verbs) is at the centre of lexical borrowing [3]. Loans may also occur in the complementary domain of function words, but these constitute a minority of borrowings. Of all lexical loans, nouns are by far the most frequent, followed by adjectives, adverbs, and verbs [4]. This captures a central function of loans: they primarily deal with items, e.g., artefacts, ideas, or notions, which in a language contact situation are impacted by socio-cultural change.

In the literature, the causalities of lexical borrowing are identified as either *need* or *prestige* [5]. Need is an internal cause, emerging out of a changing socio-cultural environment. Prestige is an external cause, where languages of more powerful cultural spheres become sources for loans in other languages. For example, languages such as Greek, Latin, German, Russian, and English are frequent loan-givers in history, depending on their socio-cultural and economic power of various periods. The fundamental principle of borrowing is that as soon as a new concept is introduced into the material or immaterial sphere of a speech community, then a designation for this new concept is needed. Alternatively, semantic change of a word can cause a gap in the vocabulary, which can be filled by means of borrowing. An example is Old English *dēor* ‘animal’, which upon changing its meaning to ‘deer’ created a slot which was filled by Latin *animal* [6]. However, languages do not need loans: the internal lexical and morphological resources are enough to coin new words. A number of “puristic” languages, such as Icelandic, illustrate this [4]. On the other hand, languages may borrow words also when there is absolutely no need for it. E.g., Domari, Otomi, or Quechua borrow frequently even within the so-called Swadesh-list, a list of basic concepts assumed (and also proved) to be exceptionally “loan-proof” [7]. The counteracting processes of borrowing and internal derivation may have multiple explanations, in which every loan event is unique. Low socio-cultural prestige may be an explanation for profound borrowing, as in the minority Romani varieties Domari or Seliče Romani [4], but low socio-cultural prestige of a speech community cannot serve as the sole explanation for high borrowability in the associated language(s). Endangered minority languages under heavy influence from a majority language may also turn out to be “puristic”, as in the Scandoromani language [8].

However, not all borrowing is random. Distinct tendencies can be observed statistically, also from a cross-linguistic perspective [4, 9]. Words from different semantic domains may differ in their borrowability: lexical items pertaining to the modern world, religion, clothing and grooming, the house, law, social and political relations, agriculture and vegetation, food and drink, and warfare and hunting, are more frequently borrowed than words from the domains of sense perception, spatial relations, body terms, kinship, motion words, the physical world, emotions, and space and time. Within these domains, just as between languages, the overall variation is extensive. Basic vocabulary lists, such as Swadesh or Leipzig-Jakarta lists, consist of concepts from the lower range of the borrowability spectrum. These lists, which are particularly popular in historical, evolutionary and cognitive linguistic studies [9–11], aim to define a cross-linguistically common vocabulary, which has a high degree of stability and robustness [12]. However, the extensive “cultural” part of the lexicon (as opposed to the “basic” lexicon) [4], i.e., the part of the vocabulary which is adapted to the culture and environment, is more complex to investigate, due to the highly varying environments of languages. The lexemes for ‘cow’ or ‘wolf’ are likely to be native to Eurasia or Africa, whereas ‘potato’, ‘maize’ or ‘armadillo’ are likely to be loanwords. In South America, we expect the situation to be the reverse [13].

The current paper targets these counteracting causes of borrowing. As in previous literature, we consider the motivations *need* and *prestige* as primary causes of borrowing [1, 2, 5, 6, 14]. These explanations can be complementary, but in a large number of loan situations, both need and prestige are present. In this sense, each borrowing situation is unique, but the aim of the current study is to test the causality of need and prestige statistically.

### Aim: Causality of lexical borrowing, studied in a coherent macro-area

The scenario described in the previous chapter serves as a background to the current study. We investigate causality of borrowing by means of empirical data in combination with statistical methods, inferred on a coherent geographic macro-area with a long history of mutual contact. A central aim is to investigate the factors of *need* and *prestige* in lexical borrowing.

The language-internal concept of *need* is difficult to define and quantify; we approach this issue from two directions. First, we look at need from a reversed angle, by looking at a section of the vocabulary where we assume the need for borrowing to be generally low. In this section of vocabulary, most borrowings would be ‟core borrowings” (a loan replaces a lexeme designating a concept previously existing in the language) rather than ‟cultural borrowings” (a loan expands the vocabulary by a new lexeme designating a new concept) [4, 15]. This represents a situation where native lexemes are replaced by loans, which can be due to internal factors, such as cultural salience and functionality, or external factors, such as contact and prestige. To investigate this, we look at semantic domains of concepts of assumed high age and stability, which we organize by an independent cultural ranking defining variation in *need*. This ranking will be further described under “Model, method and data” below.

Likewise, the concept of *prestige* is difficult to define. Prestige is a relative notion, reinvented in every instance of cultural contact, and operating in a wide range of situations ranging from interactions between two speakers to linguistic impact on a global scale. We measure prestige as the relation between languages of varying power. Our model for defining language power will be described further under “Model, method and data” below.

We focus on the domain of native culture concepts (note that our use of ‟culture” differs from other studies [4] [3]), within the domains of farming/pastoralism, hunting/war, and technology/industry. We target data that is coherent first and foremost in terms of geography, but also in terms of phylogenies. Our data also includes attested languages from previous time periods. In addition, we compare our results to a global, cross-linguistic sample. Besides need and prestige, we are also interested in the language-internal dynamics of borrowability, as well as variation in borrowability over time.

In order to investigate need and prestige in borrowability, we have defined our research questions as follows:

1. What is the general level of borrowability in our vocabulary (archaic culture concepts) with respect to languages, families, and time periods?
2. Are there any internal differences in the borrowability of lexical concepts, depending on semantic domain? How do our results relate to the average borrowability of the same lexical meaning from a global perspective? Do these differences correlate with a cultural model defining need?
3. Is there a connection between borrowability, loanword directionality and language prestige?

## Model, method and data

### The concept list

The cultural concepts in our data are selected to target lexical core concepts within the semantic domains of farming/pastoralism, hunting/war, and technology/industry, which have a recognized age of active cultural usage that stretches back at least to the Secondary Products Revolution, a the period during the Chalcolithic (4000–3000 BCE), which implied an emergence of a systematic use of secondary products of farming, such as traction or milking. Due to the long history of our selected concepts within the targeted macro-area, we assume that the words for these concepts are relatively stable over time, with a low degree of borrowability. However, we also assume that these lexemes reflect the dynamics of power and prestige, as well the dispersal of farming, pastoralism, and technological innovation during the period, which is represented by our specific selection of language families. Our targeted 117 languages (S1 Appendix) belong to the families of Indo-European, Basque, Uralic, Kartvelian, Northwest Caucasian, Nakh-Dagestanian, and Turkic, which have an estimated time-depth that varies between 7,000 and 4,000 years. These families share mutual contact over the past millennia, in several cases stretching back to the Chalcolithic period and even further. There is a rich literature on prehistoric borrowing among our families [16, 17], indicating that the mutual contact within our area stretches considerably far back in time. However, since our work is quantitative, we apply strict rules for defining loans. Our data feature many known cases of prehistoric borrowing, as demonstrated by comparative linguistics and relative chronology [5], but to avoid etymological speculation on prehistoric language contact, we measure borrowability in *attested* languages only, which also includes historical, literary languages, such as Latin and Hittite. Our earliest attested languages stretch back into the early 2^nd^ millennium BCE, meaning that there is a considerable time-gap from the Secondary Products Revolution (4^th^ millennium BCE), by which we define our concepts.

Our data includes words for predator and game animals, domestic animals, farming, metallurgy, tools, weapons, implements, and so forth. The basis for our list is a selection of core meanings, which are of primary importance to subsistence and economy (see Table 1), and which can be reconstructed to the proto-languages of the families, in particular Indo-European [18, 19]. We have extracted the dataset from the lexical subsection of the database DiACL [20], which uses a taxonomic classification of concepts (including activities, agricultural tools, crops & fruit, farm animals, products, seasons, trees, weapons, wild animals, etc.) based on the standard of the Intercontinental Dictionary Series [4, 21]. For the current study, we use a semantic classification of our data that is based on clustering of colexifications (co-occurrence of meanings of concepts) and meaning changes [22].

**Table 1:** List of concepts and their classification according to colexifying behavior.

Classification	Word List Item	Classification	Word List Item
ACTIVITIES	to plow	PREDATOR ANIMALS	bear
ACTIVITIES	to sew	PREDATOR ANIMALS	jackal
ACTIVITIES	to sow	PREDATOR ANIMALS	leopard
ACTIVITIES	to spin (thread)	PREDATOR ANIMALS	lion
ACTIVITIES	to weave	PREDATOR ANIMALS	lynx
CATTLE	bull	PREDATOR ANIMALS	snake
CATTLE	calf	PREDATOR ANIMALS	wolf
CATTLE	cattle	PREDATOR ANIMALS	fox
CATTLE	cow	PREDATOR BIRDS	eagle
CROPS	flax	PREDATOR BIRDS	owl
CROPS	grain (generic)	PREDATOR BIRDS	raven
CROPS	oats	PRODUCTS	honey
CROPS	rye	PRODUCTS	hops
CROPS	wheat	PRODUCTS	milk
CROPS	barley	PRODUCTS	salt
DOMESTIC ANIMALS	cat	PRODUCTS	wax (bees)
DOMESTIC ANIMALS	dog	PRODUCTS	wool
DOMESTIC INSECTS	bee	SEASONS	autumn
DRAFT ANIMALS	donkey	SEASONS	harvest
DRAFT ANIMALS	horse	SEASONS	spring
DRAFT ANIMALS	ox	SEASONS	summer
DRINK AND DRUGS	mead	SEASONS	winter
DRINK AND DRUGS	wine	SMALL CATTLE	goat
GAME ANIMALS	bison	SMALL CATTLE	lamb
GAME ANIMALS	deer	SMALL CATTLE	ram
GAME ANIMALS	hare	SMALL CATTLE	sheep
GAME ANIMALS	rabbit	SMALL CATTLE	kid
GAME ANIMALS	wild boar	TILLAGE	cultivated field
IMPLEMENTS	knife	TILLAGE	furrow
IMPLEMENTS	saw	TILLAGE	plow
IMPLEMENTS	scythe	TREES	ash
IMPLEMENTS	sickle	TREES	beech
IMPLEMENTS	spade	TREES	birch
MATERIALS	meat	TREES	elm
MATERIALS	leather	TREES	oak
MATERIALS	fur	WEAPONS	army
MATERIALS	grease	WEAPONS	arrow
MATERIALS	stone	WEAPONS	axe
MATERIALS	wood	WEAPONS	bow
METALS	copper	WEAPONS	shield
METALS	gold	WEAPONS	spear
METALS	iron	WEAPONS	sword
METALS	silver	VEGETABLES & FRUIT	apple
PIG RAISING	pig	VEGETABLES & FRUIT	grape
PIG RAISING	piglet	VEGETABLES & FRUIT	turnip
POULTRY	chicken	VEHICLES	axle
POULTRY	duck	VEHICLES	hub
POULTRY	hen	VEHICLES	wagon
POULTRY	rooster	VEHICLES	wheel
		VEHICLES	yoke

### Geographic area, languages and families

**Fig 1:**
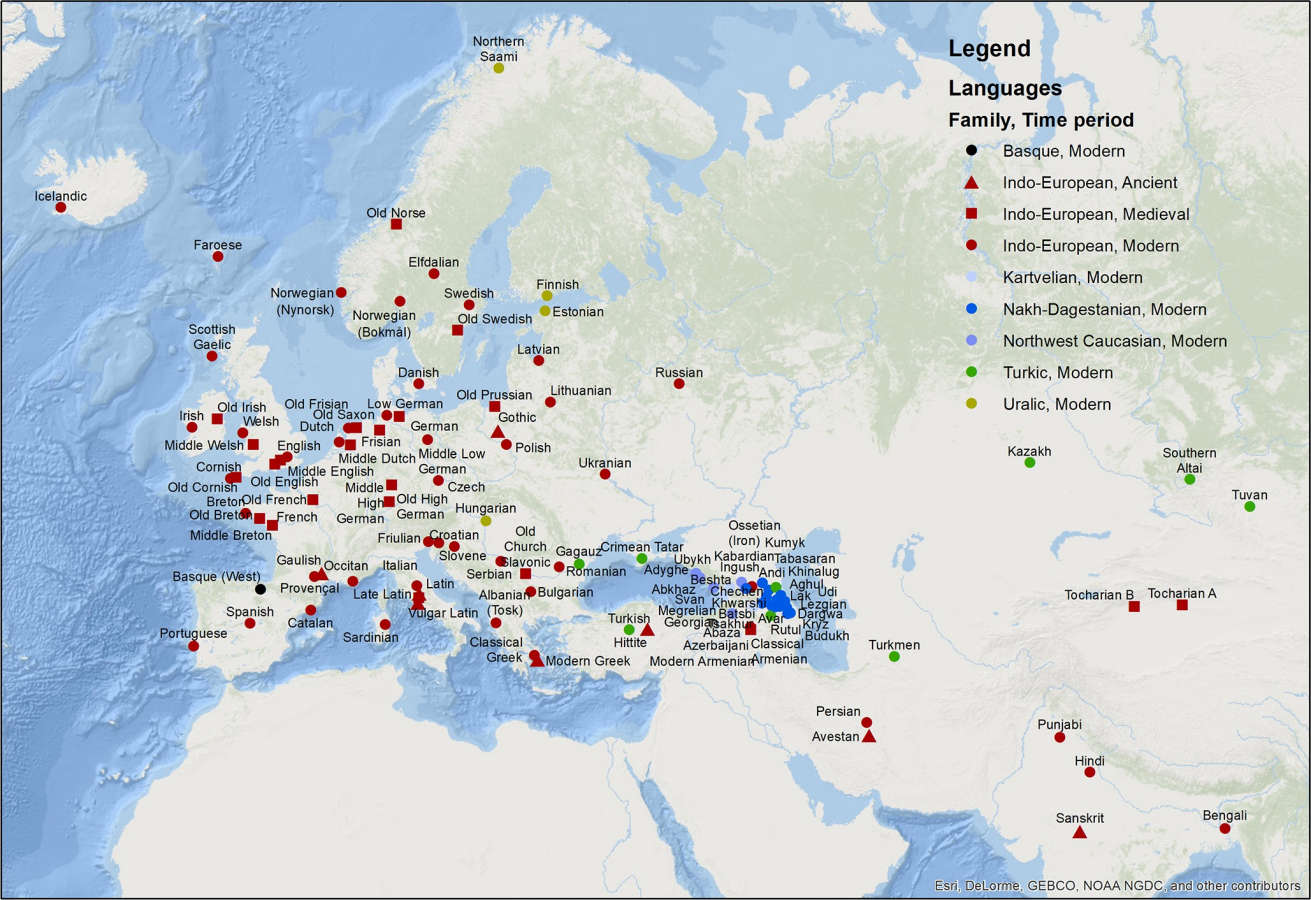
Language map. Geographic distribution of languages in the current study, defined by language family and time period, defined as ancient–(-500 ACE), medieval (500 ACE– 1500 ACE), and modern (1500 ACE– 2000 ACE).

As for our languages, we target a geographic area, which is coherent and where language contact has a long history, including (following the geographic classification by D-PLACE [23]): Northern Europe, Middle Europe, Southwestern Europe, Southeastern Europe, Eastern Europe, Caucasus, Western Asia, Middle Asia, and the Indian Subcontinent (Fig 1). The data from this area is completed by attested languages from previous periods, back to the earliest sources.

Our data includes languages from the Indo-European, Basque, Uralic, Turkic, Kartvelian, Northwest Caucasian, and Nakh-Dagestanian families. Due to their geographic location (the Caucasian mountains), the three families of Kartvelian, Northwest Caucasian, and Nakh-Dagestanian are often referred to as the ‟Caucasian families” in the text.

The selection of the geographic area, which is mainly based on the availability of data, has several implications, potentially impacting the results. Firstly, the data includes languages from families which may have genetic relatives outside of the area, leading to an uneven coverage of some families (Uralic, Turkic). Secondly, the selection results in a situation where Indo-European languages, due to the selected area as well as the frequent occurrence in historical records, are clearly dominant. We aim to account for these problems in our evaluation of the results (see ‟Discussion”). Thirdly, our data may contain lacunae, which are mainly caused by limitations in the extracted data. In particular, the coverage of Indo-Iranian languages of the Indo-European family is lower than for the remaining branches of this family.

We are aware that these shortcomings of the data may impact the results. However, since the study targets language contact and borrowability, we consider geographic coherence and mutual contact to be of higher importance than genetic affiliation. These limitations of the data also have to be accounted for when we retrieve general claims on borrowability from our results. We account for this issue under ‟Results” below.

### Coding models: Cognacy, loan status, and classification

We use a subset of the data from the lexical subsection of the database DiACL [20], which serves as an infrastructure for harbouring data analysed by the comparative method. The database organizes lexemes into cognate trees with branches, which reflect various types of change between ancestral and daughter language states, including meaning change, lexical derivation, and loan status [22]. The basis for the data is lexical core concepts, such as ox, milk, or wolf, but the cognate trees typically contain many diverging meanings, such as (for wolf) ‘murderer’, ‘thief’; (for cow) ‘woman’, ‘elephant’. Also, the coding of cognacy relations between stages represents varying degrees of certainty. For the purpose of the current paper, we have filtered and conflated some of the distinctions found in the database.

First, we have filtered out lexemes whose meaning has changed completely. Cognacy trees in the database DiACL contain lexemes with various types of meanings, including single core meanings (apple: ‘apple’), colexified meanings (apple: ‘apple; pear’), modified or specialized meanings (apple: ‘Red Delicious’ (a species of apple)), obsolete or archaic usages, and completely changed meanings (apple: ‘apple tree’, ‘forest’). We have derived a coding system [22], which specifies meanings of lexemes as: (1) core concept meaning, (2) core concept meaning (loaned), (3) secondary meaning (modified, extended, obsolete or archaic use), (4) secondary meaning, (modified, extended, obsolete or archaic use, loaned) and (0) changed meaning (not loaned/ loaned). Using this coding, we have filtered out words with semantic change (0), and used the coding (2) and (4) as a basis for identifying loans.

For our statistical analyses, we use an extract from the database, which also has additional metadata including the following information (database names are in parentheses; complete extracted data is given in S2 Appendix; the list gives fields in the order they appear in the spreadsheet):

- Lexeme ID (Lexeme ID): Database-generated lexeme ID.
- Language name (Language): Name of the language in which the lexeme is found.
- Family (Language Family): Name of the language family/stock which the language belongs to.
- Language ID (Language ID): Unique database ID of the language.
- Lexeme transcription (Word Transcription): Transcribed form of the lexeme in the language.
- Meaning (Word Meaning): Exact (from dictionary, fieldwork) meaning of the lexeme in the language.
- Word class and grammatical gender (Grammatical Data): Information on the word-class and gender of the lexeme.
- Classification in database (Classification DB Name): Classification of concept in the database, following the IDS standard (see text).
- Connected concept (Word List Item): Core concept (e.g., bull, cow, gold) connected to the lexeme in the database.
- Proto-Language Lexeme ID (Top Node Lexeme ID): Unique database ID of the lexeme of the root of cognate trees (= cognate ID).
- Proto-Language reconstruction (Top Node Transcription): Transcription of the reconstructed form at the proto-language level of the etymological tree.
- Status (Reliability): The status of the connection (Unspecified/ Inherited/ Probably borrowed/ Certainly borrowed/ Uncertain origin/ Wanderwort/ Derivation) between the lexeme and the ancestral lexeme in the cognacy tree.
- Source language (Source Language): Name of the ancestral language preceding the lexeme in the cognacy tree of the database, in this dataset given for loanwords only.

The current paper deals with words in the dataset that are borrowed, and we use a method for defining borrowability that is different from that of the Loanword Typology project database [4], as well as from the DiACL database from which we derive our data. For the statistical analyses we distinguish two coding variants, *Loan* and *No Loan*. The group Loan conflates words that have been coded as “Probably borrowed” or “Certainly borrowed” in the database, and No Loan includes both lexemes that are connected to trees (but not marked as Loan) as well as words that lack etymological information in the database.

The coding in the DiACL database is done by hand and retrieved from dictionary sources (no automation has been used at any stage) [20, 22]. The source for each cognacy and borrowing judgment in our data is rendered in the database under each lexical lemma, but this information has not been included in the attached dataset (S2 Appendix). We attach a list of the sources for cognacy and loan judgments in S1 File.

Cognacy and loan judgments in dictionaries are sometimes contradictory, in particular for historical languages. As a policy, we have used dictionaries as a source for our judgments, but the ultimate selection or decision in case of conflict is our own. We have tried to follow the standard of etymological reliability as defined by Hoffman and Tichy [24], where a reliable etymology accounts for the time and place of attestation, the frequency of occurrence, the philological environment, the word meaning and usage, the chronology and accuracy of the reconstruction of sound changes, the morphology, and the motivation for any prehistoric and historic change. In case of loans, a secure identification of the source language and the time of the borrowing depends on the sources, the etymological reliability, and the relative chronology of reconstruction of sound changes preceding and following the loan event, both in the source and in the target language [25]. Upon extraction from the database, we have checked the data to ensure that the category of lexemes not connected to trees does not contain hidden loans, following our policy and adapting to the policy of the DiACL database [22]. This implies that lexemes are coded as loans if the source language can be identified with certainty. The source language can be an attested language, a historical or extinct language, or even a reconstructed precursor (proto-language of branch or family), which can be defined in time and space. However, the source language in our data cannot be an unknown substrate language, which is frequently suggested in some etymological dictionaries [26, 27]. In cases where some dictionaries suggest loan from an unknown substrate language, whereas other dictionaries suggest that the lexeme is inherited, we code the lexeme as No Loan (see discussion below under “Results”). As a rule, we follow traditional models for the judgment of cognacy and loan coding [24, 28, 29] rather than substrate-oriented models (such as the Brill Etymological Dictionaries) [30], even though these latter resources have been an important source for the data compilation.

Another important policy, which also potentially impacts results, is the number of times a lexeme is coded as loan (see further under “Discussion”). Our data contains a number of historical languages, and thus reflects earlier stages of borrowing, which are later continued in languages by inheritance. Since we code individual loan events, we code loans only *once*, when they first enter a language. E.g., if a loan enters Old French from Latin and then continues into French by inheritance, it is coded as a loan only in Old French. However, words are often borrowed from historical languages (e.g., Latin) into later languages immediately (and in parallel), and in cases such as these, we code these target words as loans in all languages the source word has entered into. The judgments here are informed by the comparative method and relative chronology, as described before.

In the metadata coding of the database, languages are defined by their time period (from *x* to *y*). We make use of this information by adding a coding system that divides our languages into time periods. Since we use this metric for both source and target languages in our statistical analysis, and since some of our source languages are reconstructed, we make a rough division of our languages into three periods. These are ancient (all languages before 500 ACE, including reconstructed states that occur as source languages), medieval (languages between 500 ACE and 1500 ACE), and modern (all languages 1500- ACE). This division is very general, and also adapted to a Eurasian historical scenario. However, we believe that interesting information about the periodization of borrowing could be unveiled by this division, even if the exact division itself may not be immediately applicable to all geographical regions of our data.

In the database DiACL, the concepts are classified following the system used by the WOLD and IDS databases [4, 21]. We use this system to compare our results to a global sample. However, for the current study we use a different classification system based on an earlier analysis of our data [22]. This classification uses the co-occurrence of meanings of lexemes, as well as the co-occurence of the meanings of lexemes in cognacy trees, as a basis for deriving classes. Lexeme meanings are organized into meanings that co-occur within an etymological tree by co-lexification (co-occurrence of meanings by concepts) and meanings that co-occur within a lexeme by by polysemy (co-occurrence of meanings by individual lexemes) [31] by using semantic networks [32]. Based on the patterns of mutual co-occurrence of meanings, the concepts are clustered into semantic classes (Table 1), which are labelled from the OCM (Outline of Cultural Materials) classification system for cultural features [33].

Accordingly, there are three additional columns (in contrast to the database) in the dataset (S2 Appendix), containing the following information:

- Time period (additional coding, based on time stamps of languages in database): Classification of the time period of each language.
- Borrowed status (additional coding): Conflation of the statuses Probably borrowed and Certainly borrowed from the database, distinguishing Loan from No Loan.
- Classification (additional coding, based on analysis of the data): Classification of concepts based on colexification (co-occurrence of meanings in a lexeme) and meaning change (between lexemes in a cognacy tree) of the lexemes in the full dataset.

### Method: Measuring borrowability by statistical methods

As indicated in the previous chapter, borrowability can be measured along various parameters. The Loanword Typology Project [4] uses a method with an incorporated scale of five degrees of reliability (0 –No evidence for borrowing, 1 –Very little evidence for borrowing, 2 –Perhaps borrowed, 3 –Probably borrowed, 4 –Certainly borrowed), which serves to assign a borrowability value to each word, by which the results are weighted. We measure borrowability as the rate of borrowed lexemes (see “Coding models”) in relation to the total number of lexemes per lexical concept (or per language), which have kept the core meaning, also including lexemes of extended meaning and secondary usage (“Coding models”). This model serves as the basis for measuring borrowability in our study.

On our data, using the coding of lexemes and our different ranks, we perform various statistical tests. The basic statistics of levels and rates of borrowability of languages, concepts, families etc. have been performed in Excel. Correlations have been computed in R [34], and graphs have been produced by R Studio and ggplot [35]. We will describe the instruments for analysis under the corresponding sections below.

### The data in numbers

Our data integrates the culture word lists of the DiACL database for the Indo-European, Kartvelian, Northwest Caucasian, Nakh-Dagestanian, Basque, Uralic, and Turkic families [20]. The full dataset, including reconstructions and lexemes with changed meanings, originally contained 20,229 rows with unique lexemes and additional data. Of these lexemes, we remove concepts that did not have coverage in all families, which leaves us with 104 remaining lexical concepts (Table 1). We also remove reconstructed forms (of unattested proto-languages), as well as lexemes where the meaning has changed from the lexical concept meaning (e.g., ‘fruit’ instead of ‘apple’ for apple, marked as 0 in our semantic coding, cf. “Coding models”). This gives us a set of lexemes consisting of the concept meanings (including slightly changed meanings and secondary usage forms) in attested contemporary and historical languages. In the data, not all languages are filled to a satisfactory level. We decide to place a threshold of at least 30 lexemes per language for some of the historical languages, and at least 80 for modern languages. This gives us a dataset of 15,015 lexemes and 115 languages, which is the dataset that forms the basis for the current study (Table 2). The lexemes remaining after this selection process are given in S2 Appendix, ordered by language. The languages are divided into three time periods (ancient:– 500 ACE, medieval: 500 ACE– 1500 ACE, and modern: 1500 ACE–).

**Table 2:** The lexical dataset in numbers.

Families	7
Languages	115
Lexical concepts	104
Lexemes	15014
Cognate trees	1224
Loans	1516

The distribution of lexemes per time period is given in Table 3. The data includes lexemes from 7 families (Basque, Kartvelian, Northwest Caucasian, Nakh-Dagestanian, Indo-European, Turkic, and Uralic). The distribution of languages and lexemes per family is given in Tables 4 and 5. The geographic distribution of languages per family and time period can be seen in the map of Fig 1.

**Table 3:** Distribution of lexemes per time period (defined by languages).

Time period	Date	Lexemes	% of all lexemes
Ancient	500 BCE– 500 ACE	1400	9,32
Medieval	500 ACE– 1500 ACE	3117	20,76
Modern	1500 ACE –	10497	69,92
**Total**		**15014**	**100**

**Table 4:** Number of languages per family.

Family	Number of languages
Basque	1
Kartvelian	3
Northwest Caucasian	5
Nakh-Dagestanian	18
Indo-European	75
Turkic	9
Uralic	4
**Total**	**115**

**Table 5:** Number of lexemes per family.

Family	Number of lexemes	% of all lexemes
Basque	91	0,60
Kartvelian	280	1,86
Nortwest Caucasian	440	2,93
Nakh-Dagestanian	1931	12,86
Indo-European	10950	72,93
Turkic	908	6,05
Uralic	414	2,76
**Total**	**15014**	**100**

### The rankings

#### The Culture Labour Intensity rank

Our first ranking focuses on *need* and targets *concepts* and their hypothesized receptivity to borrowing. As we assume that need over time can be defined in relation to cultural salience and functionality, an important aim is to capture this by a ranking index of concepts that matches our hypotheses. Previous loan studies have indicated a strong cultural component in borrowability [4], but another study of borrowability in English and Dutch implies that grammatical category, word length, age of acquisition, and frequency may all be factors underlying borrowing rates [36]. The precise connection between borrowability and lexical replacement is not clear: the notion of ‟robustness” or ‟basicness” in basic vocabulary implies both low borrowability and lexical stability [12], but similar factors have been suggested to cause lexical replacement, such as word frequency, occurrence of synonyms, imageability, or age of acquisition [37–39]. However, preliminary tests on our data (which does not, with a few exceptions, consist of basic vocabulary, but has a presumed high age) imply a negative correlation between lexical replacement and borrowability. As we assume that cultural salience and functionality are important factors in lexical change, we formulate a primary hypothesis that assumes that borrowability increases in lexical domains representing concepts of increasing cultural involvement, where the necessity of adapting the vocabulary is higher. As indicated before, concepts representing semantic notions inherent to the environment, which are normally not changed by cultural activity, such as kinship terms, body parts, or items of the physical world, have a lower borrowability than various cultural items, which imply adaptation and change (such as clothing and grooming, food and drink, and agriculture and vegetation) [4].

For our ranking, we use a system of defining two different degrees of *need*, which we classify as low expectance of borrowing (Low = L, coding 0) and high expectance of borrowing of concepts (High = H, coding 1). We define these levels by semantic property and cultural function, from which we extract a mean value (ranging from 0–1) to classify the general expected borrowing need of a concept. In addition, we derive a subgrouping of the concepts into three classes based on the four rankings: concepts of predominantly low need (Low = L), concepts of equally low and high need (Equal = E), and concepts of predominantly high need (High = H) (S3 Appendix).

We start by dividing our concepts into two classes representing an elementary distinction: concepts that belong to the domain of *nature*, and concepts that we classify as belonging to the domain of *culture* (S3 Appendix) [40]. We hypothesize that borrowing varies within both of these classes, since concepts of both classes are more or less involved in human cultural endeavour. Therefore, we do not expect that borrowability is connected to a nature/culture distinction. Rather, we use the distinction between nature and culture as a foundation to define further rankings. Beginning with the main domain *nature*, we assume that the property of being immobile or mobile impacts borrowability. This distinction relates to the fundamental property classification of Figure/Theme and Ground in the linguistic literature [41, 42], and distinguishes nature concepts according to the property of whether they can relocate by their own (e.g., wild animals) and thus interfere with human activity (High = H), or whether they belong to the physical world and cannot relocate, a property that can be attributed to both concrete materials (metals, materials, minerals, trees) and to abstract concepts (seasons, etc.) (Low = L) (S3 Appendix).

**Fig 2:**
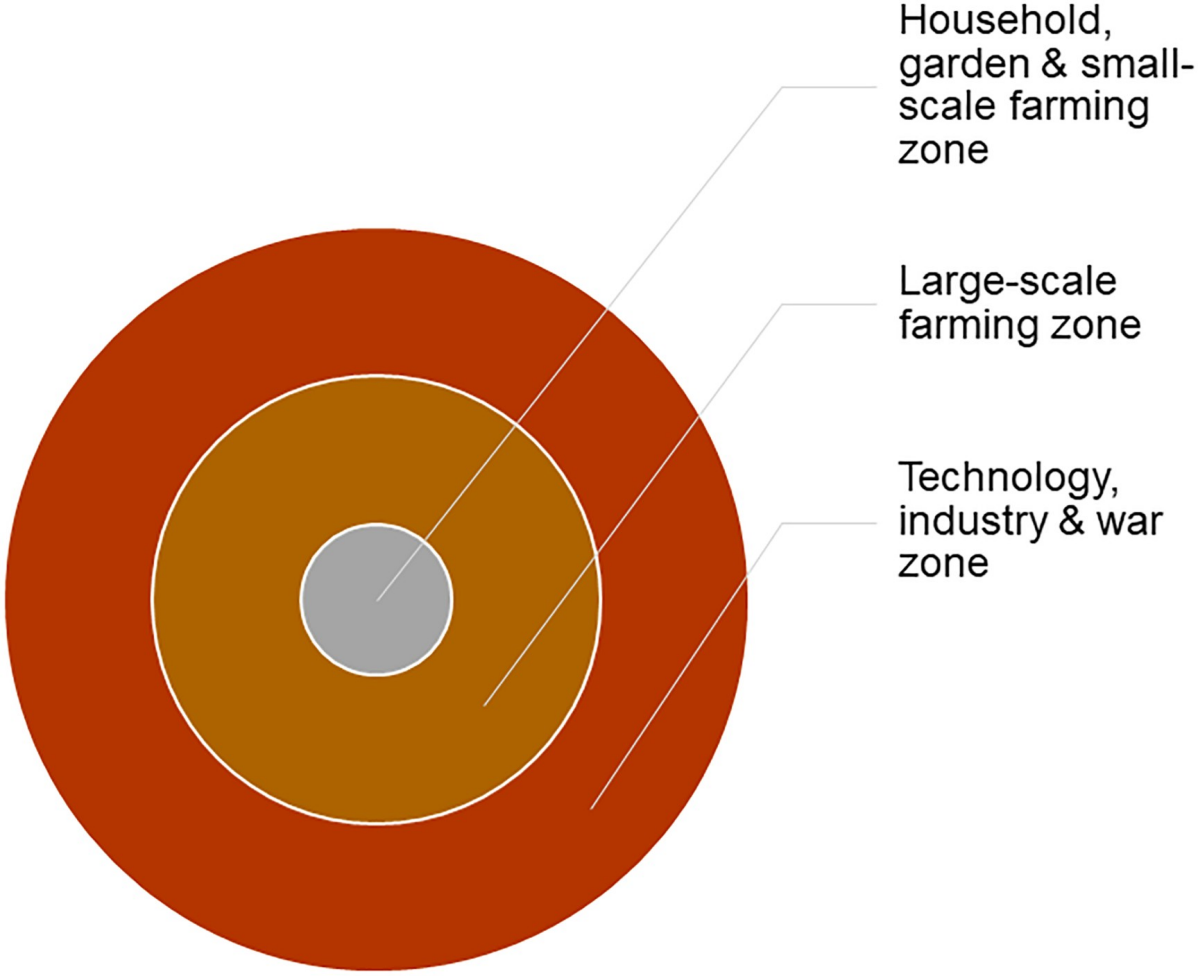
Centre-periphery model. Graph illustrating the 3 main zones of the centre-periphery model underlying the classification of concepts into our Culture Labour Intensity rank.

For the concepts of the culture domain, we adapt a spatial centre-periphery or ‟distance-from-hearth” model [43], based on the human settlement. We define these spheres as involving an increase in the intensity of cultural involvement and labour, connecting to a theory of the development of agriculture ranging from foraging and small-scale farming to large-scale farming and mechanization. This model comes from a traditional evolutionary theory of development of farming which is connected to the emergence of hierarchical tribal societies, labour division, and growth of ‟labour bottlenecks” [44, 45]. We assume, in accordance with this theory, that susceptibility to borrowing increases accordingly with increased labour intensity. Hence, we divide our model into 3 zones (from lower to higher), defined as 1) Indoor, garden & small-scale farming zone, 2) Large-scale farming zone, and 3) Technology and industry zone (Fig 2). We believe that this model is valid mainly for agricultural and pastoralist societies; we have no claims for the validity of the model on foraging and hunting populations. To investigate the validity of the model beyond farming and pastoralist societies, we would need data from languages of foraging and hunter-gatherer populations, which is currently not available.

To quantify this model by binarized expectations (H/L) as described above, we define the concepts within the *culture* domain by three different factors, which we assume imply low (L = 0) or high (H = 1) borrowability (see Fig 2, Table 6 and S3 Appendix for a complete list):

- The cultural domain type, of which we distinguish farming (including settlement) (L) and industry (H) (S3 Appendix).
- The farming type, defined as small-scale (including e.g., household, garden, small cattle) (L) and large-scale farming (H) (S3 Appendix).
- The settlement sphere, defined as household/ indoor/ garden (L) and out-door (H) (S3 Appendix).

We will look more carefully at these individual factors in relation to our results in the chapter “Causality” below.

**Table 6:** Schema of motivations for the coding of factors underlying the expected borrowability of concepts in the Culture Labour Intensity rank, illustrated in Fig 2.

Main domain:	Nature	Culture	Culture	Culture
**Factor:**	Nature: Ground-figure	General culture domain	Subsistence farming type	Settlement sphere
**Variable:** (Expectation: High (H), Coding 1)	Figure (moving objects)	Industry	Large-scale	Outdoor
**Variable:** (Expectation: Low (L), Coding: 0)	Ground (non-moving object)	Farming	Small-scale (including horticulture and pastoralism)	Indoor (including garden)

#### The Language Power Index rank

For our analysis of *prestige*, we target the borrowability rates of *languages*. Language power is a relative concept, considering that a language that is powerful in one context might be culturally, politically and economically inferior in another context. For example, we may consider the three languages Finnish, Swedish, and English. With respect to Finnish, both Swedish and English have high impact (Finnish borrows from both), but with respect to Swedish, English has higher impact, whereas Finnish has lower impact. This scenario of relative power, which reflects the dynamics of present and past linguistic and cultural interaction [14], is difficult to quantify. The difficulty increases if a diachronic aspect is included. There is a relatively rich literature on the economic and literary power of contemporary languages, which gives various metrics of size and strength [46–51]. No study adapts these rankings to historical languages. With these caveats in mind, we introduce a coding system that compares the power of languages of different periods. This power index is defined as a relative rank from 1–5 (1 = lowest and 5 = highest), which includes living, extinct, and reconstructed languages–all of which occur in our data as either target or source languages, or both. We base the index on five criteria, defined as 1) *population size* (first language speakers), 2) *economic impact*, 3) *level of colonization*, 4) *literary power*, and 5) *level of extension in prehistory*. The category population size is applied to living languages only, the three following categories are applied to all languages independent of time period, and the last category is applied to reconstructed languages only. We introduce two types of metrics, which we have distinguished by different columns in the Language Power Index dataset (S4 Appendix). The first metric is a *quantitative* ranking, which is based on quantitative studies pertaining to one of the criteria mentioned before. The second one is an *evaluative* ranking, based on literary and historical sources, which relates to one of the five metrics mentioned before. Due to the absence of quantitative data for historical languages, all rankings of these languages are evaluative.

First, we apply the criterion of population rank, which we use for living languages only, based on data from Ethnologue [52]. We divide the data into ranks between 1–5, defined as (number of first language speakers) <100 million (5), 100–50 million (4), 50–10 million (3), 10–1 million (2), and 1 million-0 (1) (Table 7). The numbers refer to census data for native language speakers (not including second language speakers).

**Table 7:** Criteria and motivations for establishing the Language Power Index.

	Population rank	Economic rank	Colonization rank	Literary rank	Prehistoric extension rank
5	<100 million	Highest	Large-scale colonizing	Global impact, large-scale translation	Language family
4	100–50 million	High	Small-scale colonizing	Local impact, lower amount of translation	Language sub-branch
3	50–10 million	Medium	Limited colonizing	Literary tradition, mostly translated	
2	10–1 million	Medium-Low	Non-colonizing	Limited literary tradition	
1	1–0 million	Low	Non-colonizing	Limited literary tradition	

As for the second criterion of economic impact, we use a combination of the ranking of the World Economic Forum’s Power Language Index [50] with other studies on economic power of languages [46, 47, 51] for modern languages. In addition, we use a Gross Domestic Product dataset from the International Monetary Fund (IMF) [53] for languages, which in general are represented by nation states (e.g., Swedish, Norwegian, Azerbaijani), and for which we lack data from the other studies. Hence, we use a combination of IMF 2017 data, with the range (GDP, nominal, US$MM) 20,000,000–1,000,000 (5), 1,000,000–500,000 (4), 500,000–250,000 (3), 250,000–100,000 (2), 100,000–0 (1) and the language economy rank by [50], with the span 1–10 (5), 10–20 (4), 20–40 (3), 40–80 (2), and 80- (1). For isolated minority languages, which are not listed in the World Economic Forum’s Power Language Index [50] and for which no independent metric of BNP is available, we put rank (1). For the evaluative rankings of historical languages, we use historical economic power as a criterion, defined as global trading (5), large-scale trading (4), medium trading (3), low trading (2), and limited trading (1). We admit that the distinctions between (3) and (4) are difficult, in particular since economic power and trade are tightly connected to the third criterion, colonization.

The third criterion, level of colonization, is even more complex, in particular when comparing the contemporary and historical languages. The issue connects to linguistic imperialism, for which there is a rich critical literature, mostly on the role of English as a global language and the situation of minority languages in education and science [54, 55]. We find no quantitative data for this metric, and therefore, all distinctions are evaluative (S4 Appendix). In order to enable a comparison between languages of different periods, we divide them into 4 groups: large-scale colonizing (5), small-scale colonizing (4), colonizing to a limited extent (3), and non-colonizing (2–1). Adapting these ranks to extinct languages is tricky, in particular since the scope and extent of colonization have expanded significantly in the last centuries. In earlier periods, expansion and colonization by land was more frequent than it is today [56]; an example is the hugely impactful Hittite empire during the 2^nd^ millennium BCE [57]. We evaluate the relative extension of colonization in terms of geographic extension and distribution of colonisation, amount of settlements, duration and maintenance of settlements, and local impact and historical role of settlements.

The fourth criterion, literary power, is relevant to modern languages, where large-scale corpora of social media, newspapers and literature are available. Metrics, such as number of published books, tweets, published online articles, translations, etc. can be measured and analysed statistically, defining the amount of literature as well as hierarchies of translation [49]. The tricky issue is to transfer these hierarchies to extinct languages. We use the *amount* of literature together with the *frequency* and *direction* of *translations* as our main criteria. Fundamentally, the most powerful languages have the largest total amount of literary works, they are most frequently translated into other languages, and they are involved in the translation of literature from other languages; powerful languages are both receivers and givers of literary impact. Transferred to historical languages, the most powerful languages typically have larger amounts of preserved texts, due to the ways in which texts often are preserved: through literal transition. Just as with modern languages, the translation process is hierarchical, with the most powerful languages at the top, and the least powerful languages at the bottom. We have decided to code a language with a canon that is translated into a large amount of other languages, distributed over vast areas, as (5). A language with still high numbers of languages into which the canon is translated is coded as (4), whereas languages with a literary canon that is basically a translation from a language of type (4) and (5) is coded as (3). As (2) we code languages with a limited or fragmentary literary tradition. We use two different metrics: a quantitative metric for living languages, which we base on data from Ronen et al. [49], and an evaluative metric for extinct languages, defined by the amount of literature and frequency of translations (described above). The data by Ronen et al. is based on the amount and the co-occurrence (users, editors, translations) of languages in book translations, Wikipedia and Twitter. Most of the contemporary languages in our corpus are included in Ronen et al. study (including some historical ones, but we did not use this data for our rankings). In particular, we consider book translations, which we regard as the most reliable and detailed metric (as opposed to Twitter and Wikipedia), and which can be compared to our metric for historical languages. The result of the Ronen et al study is given as a hierarchical network, where the more powerful languages in terms of book translations (marked with arrows) are located at the centre. We use the following distinction to transfer this network to our rankings: languages with the largest bullets (indicating high occurrence of literature) at the centre of networks, with arrows both *from* as well as *to* at least one-third of the other languages, are set to (5). In our case, this is only valid for English and Russian. Languages with large bullets at the centre of networks, but with most arrows leading *to* rather than *from* are set to (4). Languages with relatively large bullets, but with fewer arrows, and arrows leading mainly *to*, are set to (3). Languages with occasional arrows in both directions are set to (2) and languages with arrows only in the direction *to* are set to (1). The rankings are found in S4 Appendix.

However, it is important not to put too much focus on literary tradition. Our knowledge of languages of the past is confined to languages with preserved literature. On the other hand unwritten source languages can be identified by the comparative method and relative chronology. We have several languages of this type in our data (see “Coding models”). An example is Proto-Turkic, which is not attested, but which is highly frequent in our data as a source for lexemes in the Caucasian families. Likewise, Proto-Iranian is an important source language for several lexemes in the Uralic languages. Both of these languages are unattested, but can be safely reconstructed by the comparative method. To create a compatible rank for reconstructed languages is an impossible task. However, we have decided to consider the ability to diverge and expand as a mark of relative strength, and therefore we have added a coding system which defines proto-languages of families as a (5) (which makes, e.g., Proto-Indo-European equal to Russian and English), proto-languages of relatively wide-spread branches as (4) (which makes, e.g., Proto-Germanic and Proto-Romance equal to Hindi and Bengali), and proto-languages of less wide-spread branches as (3) (which makes, e.g., Proto-Circassian equal to Danish).

The different ranks (Table 7) are weighed against each other to come up with a final rank number, by calculating a mean value. We reduce the mean value to one decimal (e.g., 4,8). The full dataset for our Language Power Index is given in S4 Appendix.

## Results

### Borrowability rates

#### Borrowability by language and language family

Our first question targets the level of borrowability in general, with respect to languages and language families. Before presenting results for this measure, a few words should be said about the comparability of the data in our corpus.

Our corpus, even though it is extensive enough for reliable statistical conclusions, is partly heterogeneous. This is due to various factors, of which the first and most important is the different status of the languages with regards to previous research. Many of our languages, particularly within the Indo-European family, are very well-researched: dictionaries are based on extensive literary corpora and large numbers of speakers. Other languages have limited, few, or no dictionaries, and the data for these languages in our corpus is based partly on fieldwork, and partly on existing dictionaries [22]. This means that the data from a language such as Udi or Khinalug is based on a handful of speakers, whereas the data from larger languages may be based on millions of speakers. Second, our languages are very different in terms of the level of etymological research. Whereas the origins of the lexicon in the Indo-European, Turkic, and Uralic language families is almost completely understood, families such as the three Caucasian ones (Kartvelian, Northwest Caucasian, Nakh-Dagestanian) are underresearched in comparison. A majority of the lexemes in these languages have uncertain etymologies. However, this does not make it impossible to distinguish loans from non-loans. In the Caucasian families, there is a high degree of loans from, e.g., Turkic languages and Arabic, which can be identified with certainty.

As described under “Method: measuring borrowability by statistical methods”, we have two basic types of lexemes in the database: lexemes that are connected to cognacy trees (either as borrowed or inherited), and lexemes that are not connected to cognacy trees. We have recoded and reworked all of the retrieved data, and checked both of these types for borrowings. The result is a coding system that distinguishes Loan and No Loan (S2 Appendix). The absolute numbers and the percentage distributions of these types, with respect to languages and types, are found in S5 Appendix. We measure borrowability as the rate of Loan in relation to No Loan. In order to understand the results, explanation and breaking down of the statistics is necessary.

**Fig 3:**
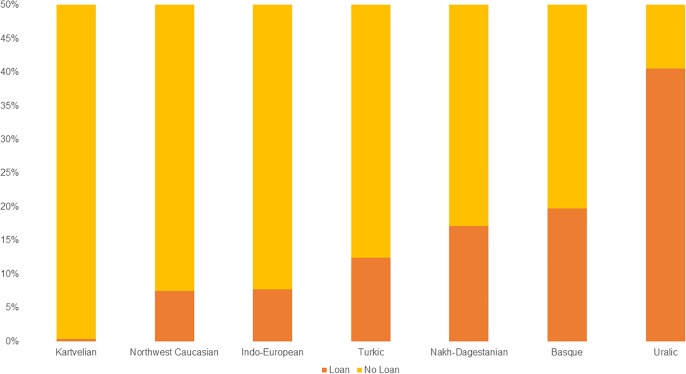
Borrowability by family. Bar plot indicating the average percentage of Loan and No Loan in the data, by language family.

**Fig 4:**
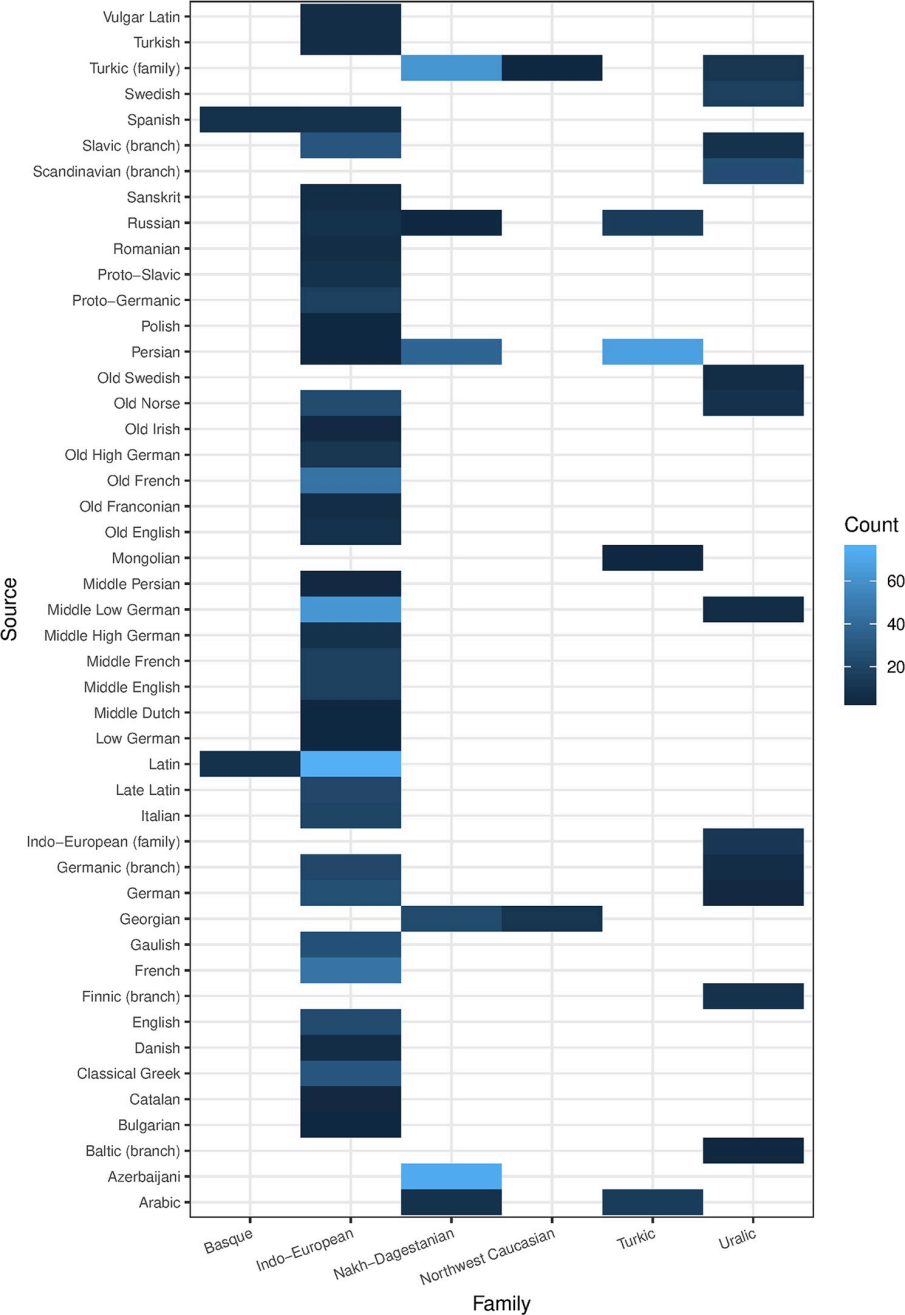
Source languages by family. Heat map of frequency of source languages in loan events with more than 5 occurrences, distinguished by language family.

To begin with, the total average rate for Loan is 10,10%. When this result is split up by language family, we see that the average rate for Loan is 19,78% in Basque, 0,36% in Kartvelian, 17,14% in Nakh-Dagestanian, 7,50% in Northwest Caucasian, 7,78% in Indo-European, 12,44% in Turkic and 40,58% in Uralic (Fig 3). As mentioned before, there is a small risk that the No Loan category of Caucasian families contains hidden loans, due to the fact that these languages are lesser researched for etymology compared to the other languages of our corpus, and sources of loans cannot always be identified with certainty. The Turkic and Uralic results are based on fewer languages, and are not fully representative of their families. The average level of borrowability in Uralic is high, which can be explained by the fact that the vocabulary for farming and pastoralism was borrowed from Indo-European, a well-known fact that is reflected in our data [58]. For Indo-European, the high availability of data from historical languages possibly affects the results in relation to the other families (see Table 3). The way in which we code data implies that a loan is coded only *once*–when it is originally borrowed. E.g., if a lexeme is borrowed from Gaulish into Latin and then inherited into all Romance languages, it is coded as Loan in Latin only, not in all Romance languages. This situation is not transferable to the other families, which lack ancient attested language stages. A loan from Proto-Iranian in Finnish and Estonian is marked as loan in Finnish and Estonian, even if the lexeme may have entered the language at an early state. To overcome this unavoidable inconsistency, we break the family data down by several parameters. The first parameter is source language. For Indo-European languages, there are multiple different source languages, both reconstructed, extinct and contemporary (Fig 4, S2 Appendix). For Nakh-Dagestanian and Northwest Caucasian (Kartvelian has very low loan scores in our data), we notice that a couple of source languages dominate, in particular Turkic (family), Persian, Georgian, and Azerbaijani (Fig 4). Evidently, these loans represent different stages of borrowing in these families. For the Uralic languages, we can also identify borrowings in several layers, from the very early proto-languages Indo-European, Turkic (family), and Indo-Iranian (branch), to the Baltic, Scandinavian, and Slavic branches, over the medieval languages Middle Low German, Old Norse, and Old Swedish, to the modern languages Russian and Swedish (Fig 4). Hiding underneath the relatively low borrowability of Indo-European we have all potential prehistoric and substrate borrowings, which are not counted as loans according to our model [28, 29, 59–61]. We notice that the most frequent source languages in Indo-European are Latin, Middle Low German, French, and Old French (Fig 4).

Besides these family-related differences in borrowability, there are also large differences in borrowability among the individual languages in the data (see S5 Appendix).

#### Borrowability by grammatical type and semantic domain

Our data is based on 104 core concepts (Table 1). In this chapter, we examine the variation in the borrowability of concepts (S1 File). Of our concepts, 5 are verbs, and the remaining concepts are nouns. The mean borrowability of the 5 verbs is 1,3%, while the mean borrowability of nouns is 11%, thus confirming the expectation that verbs have lower borrowability [4].

**Fig 5:**
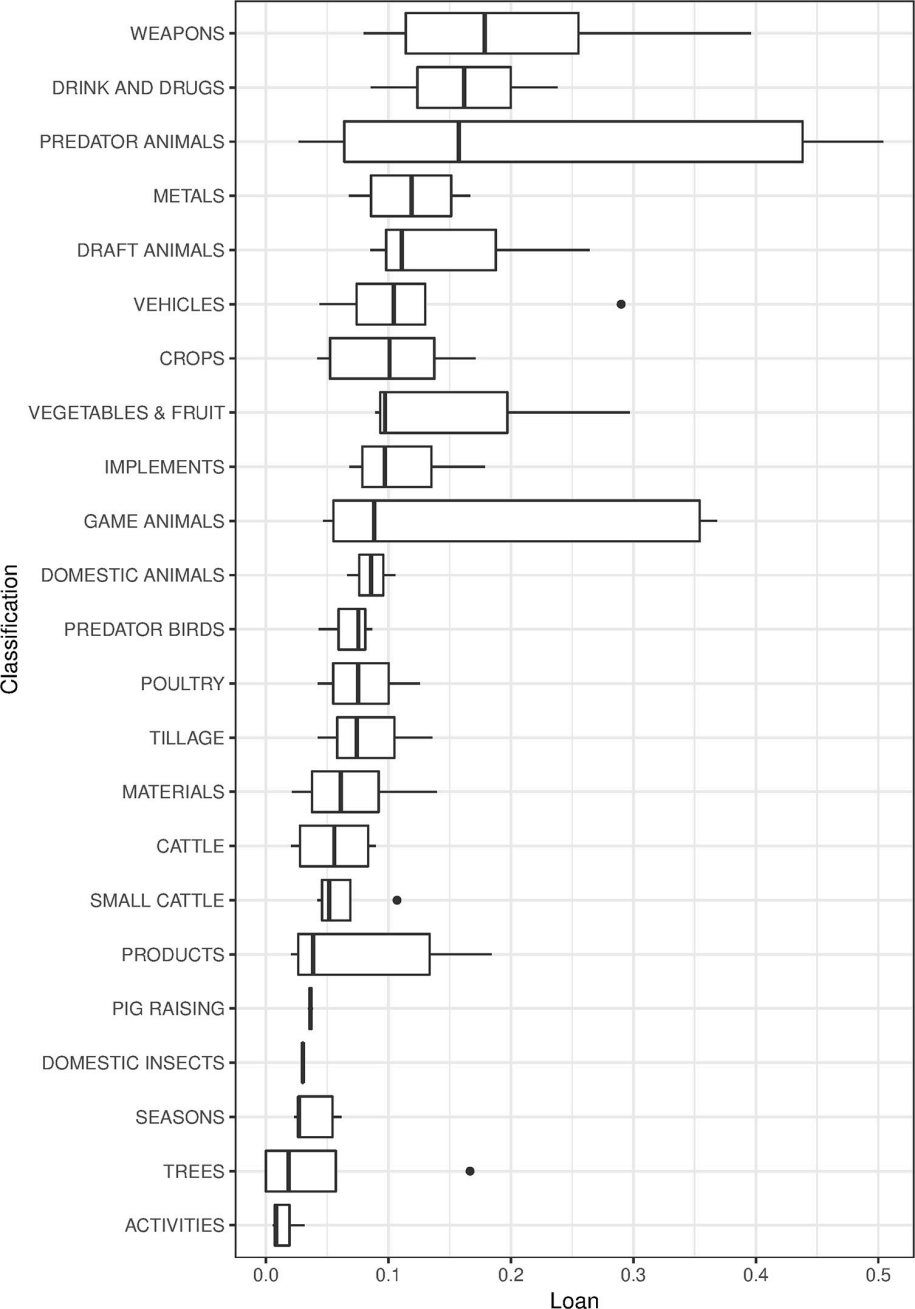
Borrowability of concepts. Boxplot showing the distribution of borrowability rates of concept classes, defined by colexification and meaning change.

We test the level of borrowability of concepts according to the semantic classification based on colexification and change (Table 1). The results, sorted from lowest borrowability to highest, are shown as a boxplot in Fig 5. The high level of borrowability in predator animals can partly be explained by the territories of our targeted predators, which are to a larger extent restricted to climate zones. However, this is not the case for the game animals (deer, hare, wild pig etc.), which have a much more extended natural range (but with variability in different species) [62]. This may partially explain the high level of borrowability of predator animals in contrast to game animals, but it cannot explain why both groups score higher than, e.g., domestic animals. The groups with above average borrowability (predator animals, weapons, draft animals, drink & drugs, vegetables & fruit, game animals, metals, vehicles, and implements) belong to various semantic and cultural domains, according to our classification of concepts as high (H), medium (M) or low (L) expected borrowability (see “The Culture Labour Intensity rank”). The groups below average (crops, domestic animals, tillage, poultry, materials, predator birds, cattle, trees, products, small cattle, pig raising, seasons, and domestic insects) belong (with a few exceptions, e.g., predator birds) either to in-door activities or small-scale farming. The result point to an increase from low to high borrowability which matches our cultural model (see further below under “Causality”).

#### Borrowability by time period

**Fig 6:**
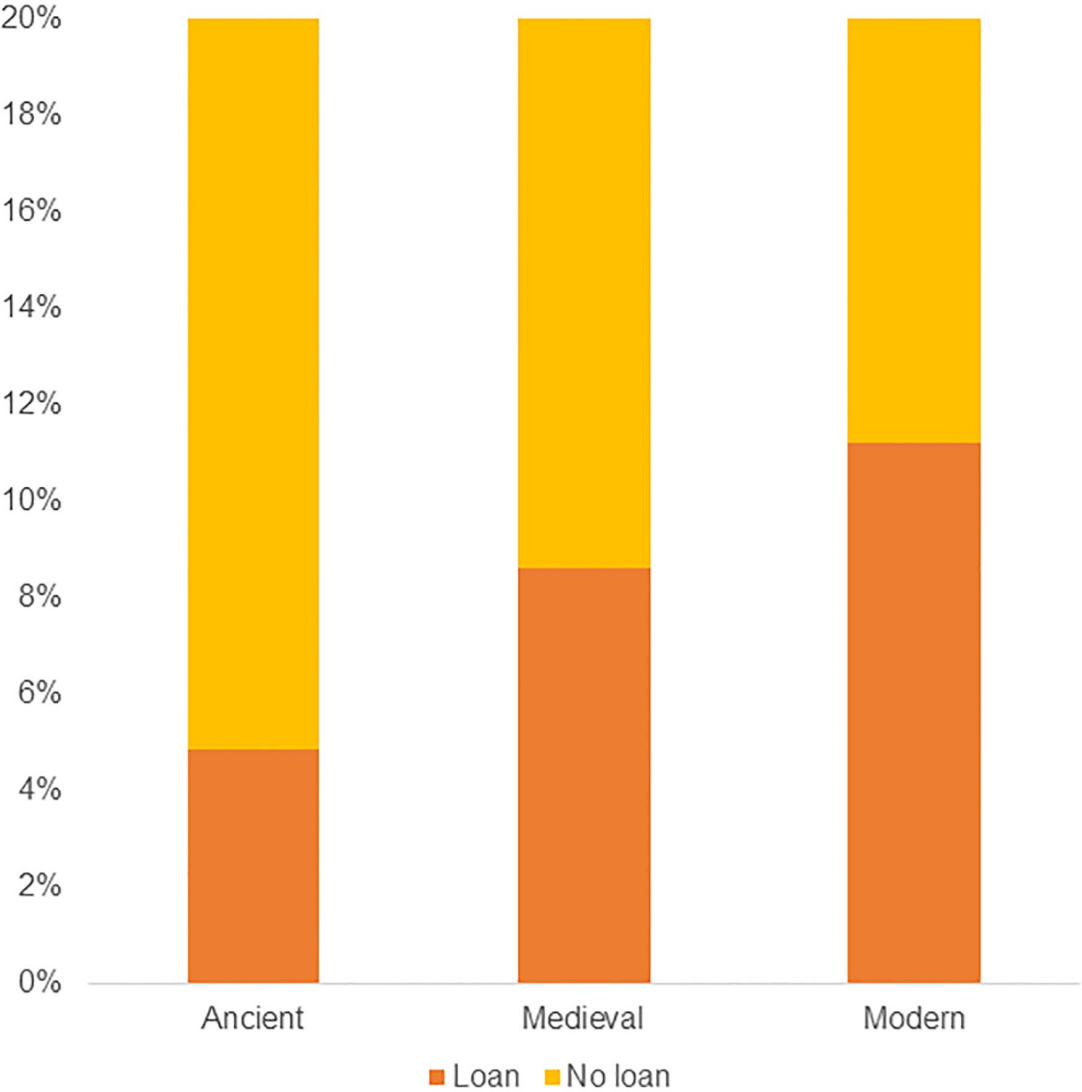
Borrowing by time period. Average percentages of Loan and No Loan in relation to the total amount of lexemes per time period (ancient: -500 ACE, medieval: 500–1500 ACE, and modern 1500- ACE).

Our data shows a slight increase in borrowability over time. The amount of data is consistently increasing over time, with 9,32% of the data from the ancient period–(-500 ACE), 20,76% of the data from the medieval period (500 ACE– 1500 ACE), and 69,92% of the data from the modern period (1500 ACE– 2000 ACE). This is expected; data amounts and language attestations increase the closer they are to present time. Our measure of increase in borrowability is calculated in relation to the total amount of data for that period (Fig 6).

We see a slight increase in borrowability over time, with 4,86% in the ancient period, 8,63% in the medieval period, and 11,23% in the modern period. As described under “Method: measuring borrowability by statistical methods”, we code lexemes as loans only if the source language can be identified. This policy excludes all assumed substrate loans, where the source language is unknown (they are coded as No Loan in our data). At least in Indo-European, there is a discussion on substrate etymologies, where parts of the Germanic and Classical Greek vocabulary are assumed to be borrowed from an unknown language (see further under “Discussion”) [63].

#### Comparison between our results and a global sample

As our data sample only accounts for a limited geographic area, we compare our borrowability scores to those of the Loanword Typology Project (LTP) [4] to see how they compare to a study with a global scope. The LTP study contains fewer languages (41, compared to our 115) but a larger number of lexical meanings (1460, compared to our 104). In addition, their languages are deliberately sampled from across the globe, to minimize any genetic or areal effects. Moreover, their meaning list is constructed to include concepts from a variety of semantic domains, with the ultimate goal of proposing an empirically based basic vocabulary list of high stability and robustness (see “Background: lexical borrowing”). Our study, on the other hand, focuses on a selected list of lexical domains in a delimited, continuous geographical area (see “The concept list”).

The LTP study uses a weighting model where loanwords are assigned a heavier weight the more reliable their loan etymologies are. To ensure compatibility with our study, which uses no such weighting principles, we recalculate the LTP scores into basic percentages. We also limit our scope to the lexemes that overlap in both studies, leaving us with a list of 84 lexical concepts.

**Fig 7:**
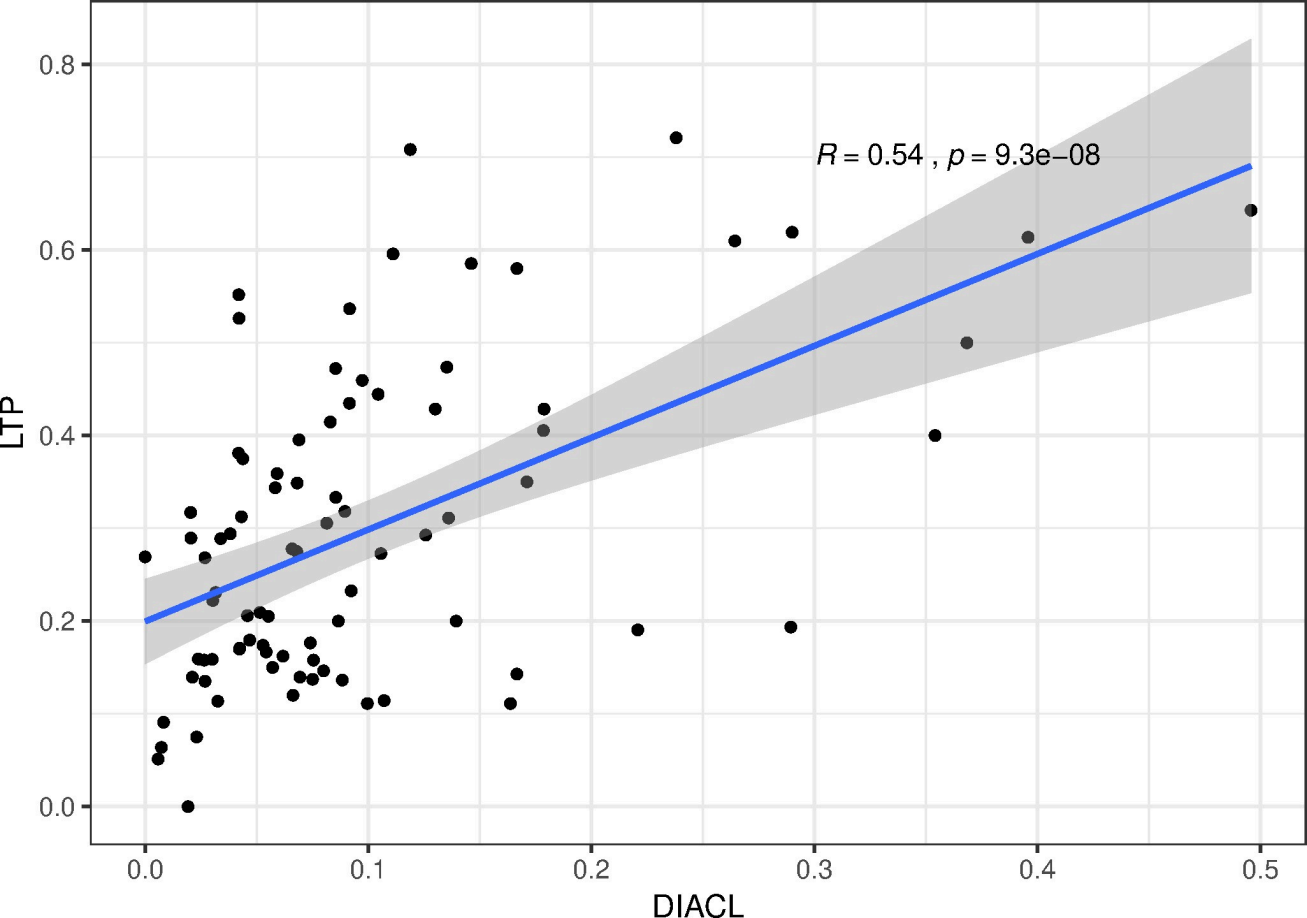
Borrowability of concepts against a worldwide sample. Correlation of rates of borrowability of concepts of our data (from the DiACL database), against a worldwide sample (the WOLD database) (Spearman’s rank *R =* 0.54, *p* = 9.3e-08).

The average rate of borrowability is much higher in the LTP study (29,80%) than in ours (10,10%). This is in line with the expectations, as we have targeted concepts that are native to the Eurasian cultural environment, but typically borrowed outside of Eurasia. However, the correlation between the concepts’ borrowability rates is significantly positive (Spearman’s rank *R =* 0.54, *p* = 9.3e-08), indicating that our general results are also relevant from a global perspective (Fig 7).

### Causality

#### Need: Borrowability in relation to the Culture Labour Intensity rank

**Fig 8:**
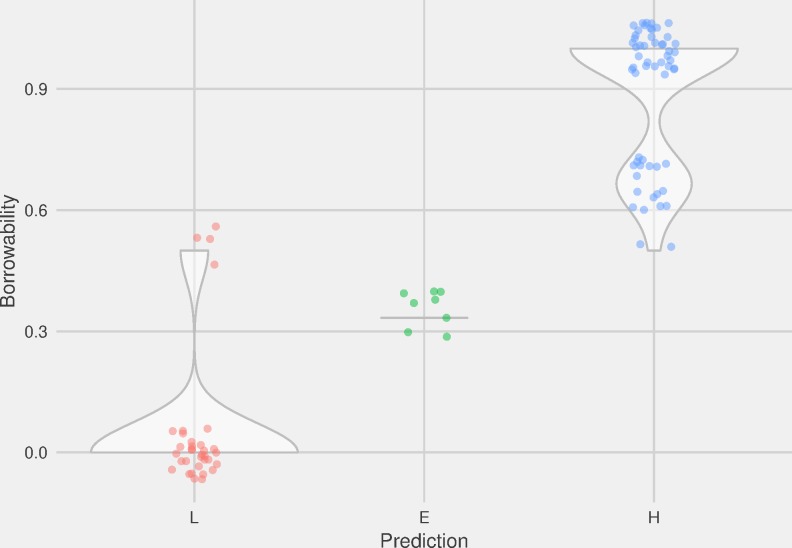
Borrowability of concepts against *need* expectations. Violin plot of distribution of borrowability of concepts within the expectation classes L (Low), E (Equal), and H (High) of the Cultural Labour Intensity rank, defined by the binary expectations (Low<High) of the variables Ground<Figure, Farming<Industry, Small-scale<Large-scale, and Indoor<Outdoor.

Our first question concerns the need for borrowing. We expect that borrowability will increase with increased cultural labour intensity, which we define by a ranking system using binarized expectations (Low = L or High = H), for five different factors (Low<High): Word class: Verb<Noun, Nature: Ground<Figure, Culture domain: Farming<Industry, Subsistence farming type: Small-scale<Large-scale, Settlement sphere: Indoor<Outdoor) (see “The Culture Labour Intensity rank”, Table 6, and S3 Appendix). We use the binary values of L = 0 and H = 1 to assign a mean value for each lexical concept, ranging from 0 to 1. In addition, we compute an overall ranking for the concepts, according to whether the score is below average (L), average (E) or above average (H) (S3 Appendix). First, we test the level of borrowability of our concepts against the mean ranking index, using Spearman’s ranked correlation (due to the nature of the data as ordinal). We find a positive correlation (*R =* 0.32, *p =* 0.0009) between the mean rank and borrowability, confirming our hypothesis that borrowability in culture vocabulary increases with increasing labour intensity. However, the variation in the distribution of the data points is also worth noticing. This is seen in in the violin plot of Fig 8, which shows the spread of borrowability of concepts of the groups of Low (L), Equal (E), and High (H) expectation. In group L (Low), a majority of the data have low values, and only a small number of items go against the prediction. Group E (Equal) has a very narrow density, whereas group H (High) has a bimodal distribution with the highest values.

**Fig 9:**
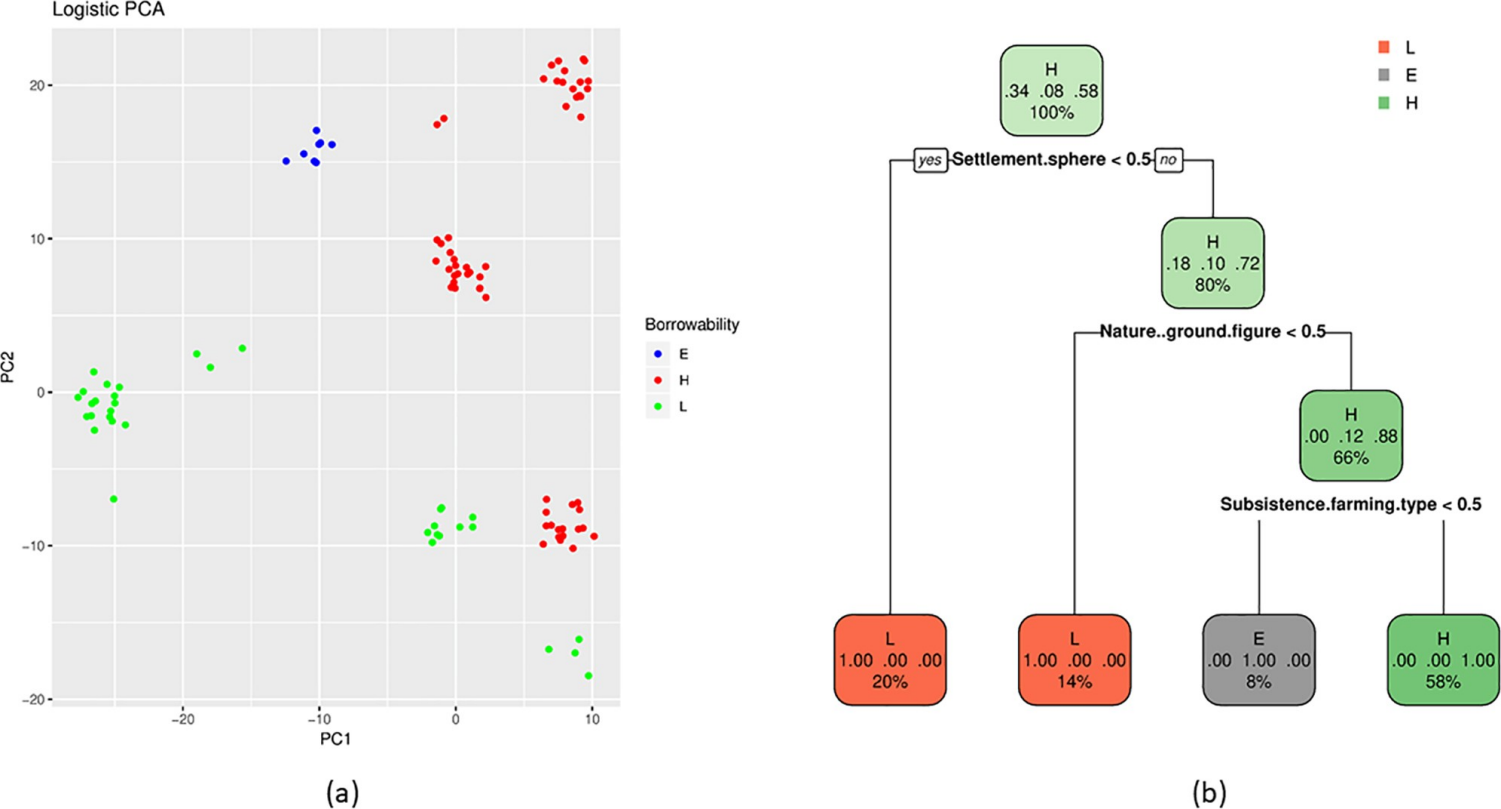
Evaluation of factors for need expectations. (a) Principal component analysis (PCA) plot of the distribution of borrowability of concepts in relation to the need expectations L = Low, E = Equal, and H = High of the Cultural Labour Intensity rank. (b) Classification and regression tree (CART) validating the individual factors of the Cultural Labour Intensity rank (Ground<Figure, Farming<Industry, Small-scale<Large-scale, and Indoor<Outdoor) for the expectation classes L = Low, E = Equal, and H = High.

We test our expectations, using the factor variables Ground<Figure, Farming<Industry, Small-scale<Large-scale, and Indoor<Outdoor. We leave out Verb<Noun due to the low number of verbs in our data. For a start, we perform a Principal Component Analysis (PCA). The primary objectives of PCA can be summarized as a) to discover or to reduce the dimensionality of the data set and b) to identify new meaningful underlying variables. PCA for binary data, known as logistic PCA, has become a popular alternative to dimensionality reduction of binary data. We use the R package logisticPCA [64]. Using the formulation in this package, a concept (represented by its variables; a feature vector) is approximated in a two-dimensional latent space. Fig 9A shows the PCA scores for the concepts, coloured by their concept label (L = Low, E = Equal, and H = High). We see that the procedure does a good job of separating the concept labels based on their variables.

We also perform a classification and regression tree (CART) analysis on our concepts. This is a method that is used to construct decision trees automatically from data. Decision trees are a ranked set of yes-no-questions. A CART is a statistical model, which can deal with incomplete data and multiple types of features both in input features and predicted features. In a concept borrowability context, the input features are the chosen factor variables of the concepts (Table 6, S3 Appendix) and the output features are the designated borrowability. The rules that are produced are relatively human-readable since they are formulated in terms of questions about the data. To perform this test, we use the R package rpart [65]. The rpart package builds classification or regression models of a very general structure using a two stage procedure; the resulting models can be represented as binary trees. The process of building a tree is described in the rpart manual as such: “first the single variable is found which best splits the data into two groups (‘best’ will be defined later). The data is separated, and then this process is applied separately to each sub-group, and so on recursively until the subgroups either reach a minimum size (5 for this data) or until no improvement can be made.” Since our primary goal here is to use decision trees for exploring and describing the data, we do not perform any training/testing split. Instead we use all available data when constructing the trees.

The performance of a tree is measured by its accuracy, which is the proportion of correctly assigned labels (H = High, E = Equal, and L = Low) that a given tree assigns to a concept. The resulting tree is shown in Fig 9B, where we see that three simple rules are able to describe all of the data. To test the stability of our designated factors, we perform a validation test, where we rebuild the trees, throwing out each of the four component factors (see S2 File). When removing the factor “General culture domain” (Farming<Industry), a tree based on the remaining factors produces an accuracy of 1, meaning that this factor does not add any information that is not covered by the other factors. When removing the factor “Subsistence farming type” (Small-scale<Large-scale), the remaining factors produce an accuracy of 0.92, when removing “Nature: Ground-Figure” (Ground<Figure), the remaining factors produce an accuracy of 0.86, and when removing “Settlement sphere” (Indoor<Outdoor), the remaining factors produce an accuracy of 0.73. This gives us a ranking of an order of importance of the factors as “Settlement sphere” < “Nature: Ground-figure” < “Subsistence farming type” < “General culture domain” (see S2 File). Additionally, we test to remove all factors except for one, to give an estimate how each factor performs in isolation. The result is given in Table 8.

**Table 8:** Accuracy of the different factors of the Cultural Labour Intensity rank given as percentage of correctly labelled concepts as regards to borrowability (cf. Table 6).

Main Domain:	Nature	Culture	Culture	Culture
**Factor:**	Nature ground-figure: (Ground<Figure)	General culture domain: (Farming<Industry)	Subsistence farming type: (Small-scale<Large-scale)	Settlement sphere: (Indoor<Outdoor)
**Accuracy (%)**	73	47	51	78

The results support the theory of labour intensity as a causality to increasing borrowability; in particular, the strength of the “Settlement sphere” (Indoor<Outdoor) factor is an argument in favour of this approach. However, the poor result of the “General culture domain” (Farming<Industry) factor is also somehow expected: both are high-labour activities of the cultural domain.

#### Prestige: Borrowability in relation to Language Power Index

**Fig 10:**
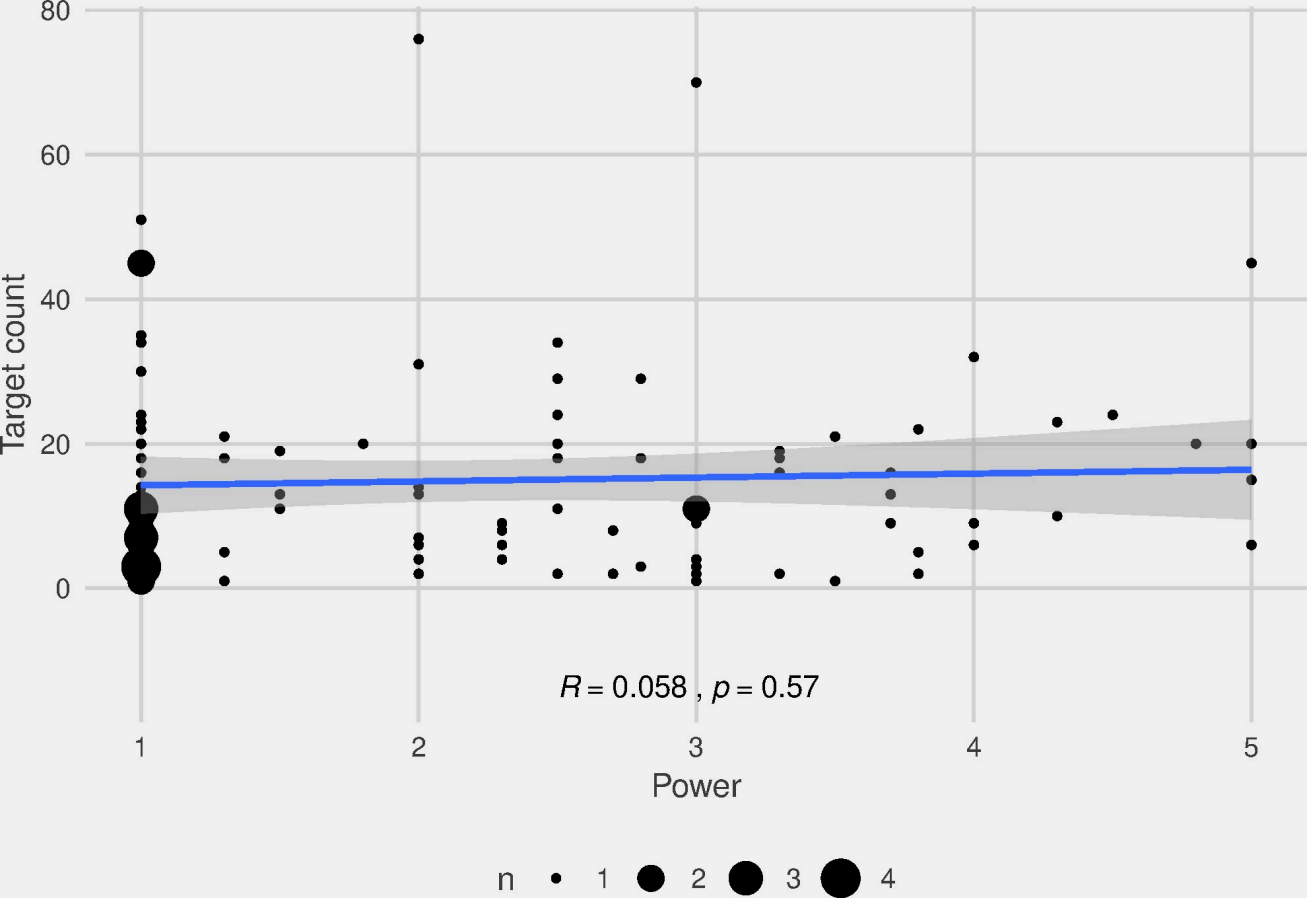
Correlation between LPI and target language of loan events. Scatterplot showing the correlation between the Language Power Index rank and occurrence as target language in loan events.

To test prestige, we use our data on loan events, each involving a source and a target language, and our Language Power Index (LPI) rank of languages ranging from 1 (least powerful) to 5 (most powerful). The issue of language power and borrowability involves directionality and requires a study of both source and target languages. Potentially, both powerful and weak languages borrow lexemes. Hence, we do not expect a significant correlation between LPI rank and level of borrowing or the occurrence as target language in loan events. This proves to be the case: The correlation between LPI rank and occurrence as target language in loan events is not significant (*R =* 0.058, *p =* 0.57, Fig 10).

What is more likely to be relevant is the directionality of borrowing: we predict prestigious languages to be more frequent as source languages in loan events. The issue is two-sided; the fundamental point is to figure out which language borrows from which language in relation to their positions within the LPI rank. We measure this using two parameters: 1) the correlation between LPI rank and the frequency of occurrence as source language, and 2) the directionality frequency of source and target languages in relation to the LPI rank.

**Fig 11:**
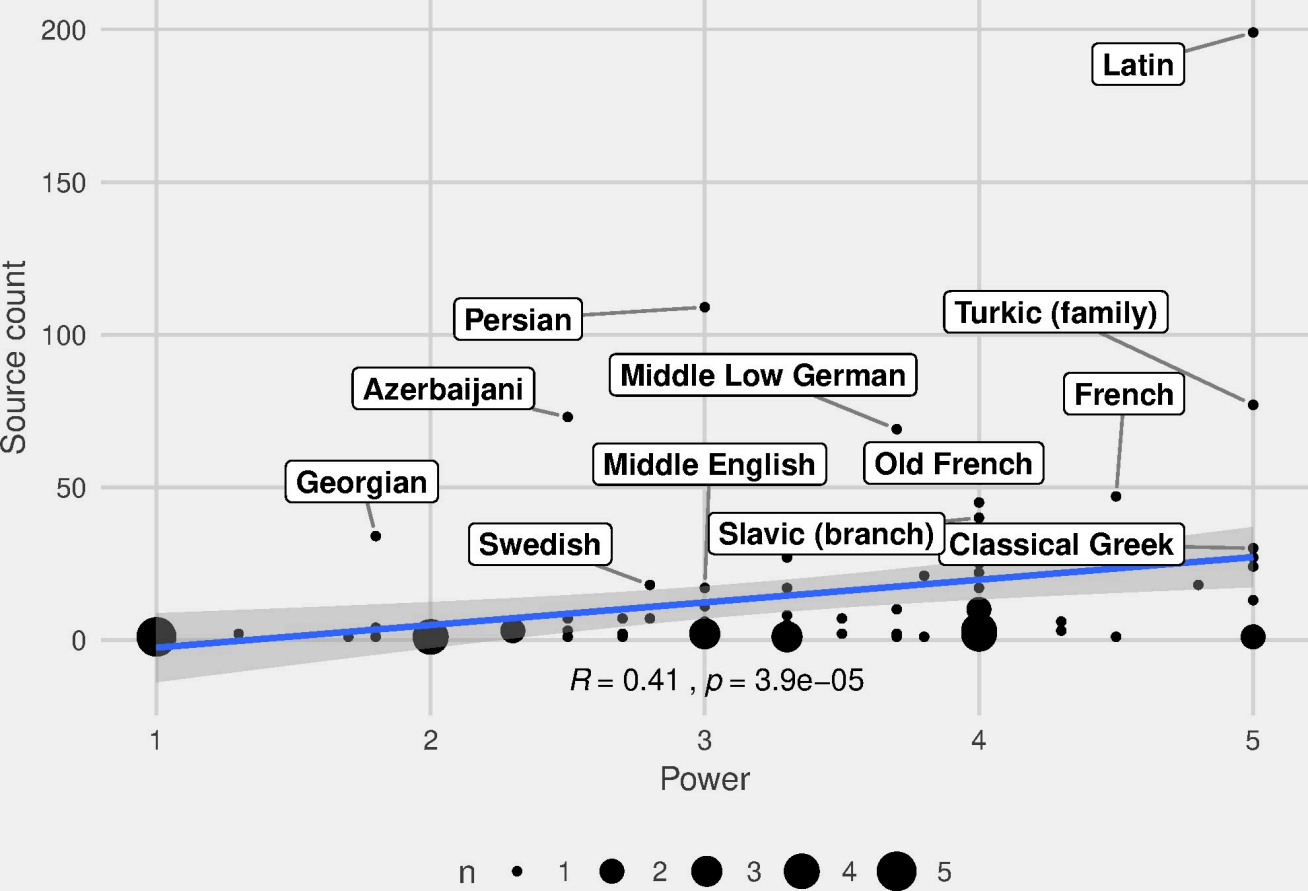
Correlation between LPI and source language of loan events. Correlation between the Language Power Index rank and occurrence as source language in loan events.

Beginning with occurrence as source language and LPI rank, we note that the correlation is positive, not exceptionally strong, but clearly significant (*R =* 0.41, *p* = 3.9e-05). In the plot (Fig 11), we may identify some interesting data points, including Latin, which is exceptionally frequent as source language and very powerful (LPI rank: 5,0), and has an expected position in the data. A few other languages have unexpected positions, which can be explained by their relation to other languages in our corpus: Georgian and Azerbaijani, which score relatively low in the LPI rank, are frequent source languages in the other languages of the Caucasus region. Other noteworthy languages in the data are Persian, which is frequent as a source language in many eastern languages (in particular in the Caucasian families), and Middle Low German, which is frequent as a source language in European languages. In fact, the source languages (apart from Latin) are more evenly distributed among European languages, whereas in the Eastern area, a handful of languages stand out as frequent source languages (Proto-Turkic, Persian, Azerbaijani, and Georgian) (Fig 4, S2 Appendix).

**Fig 12:**
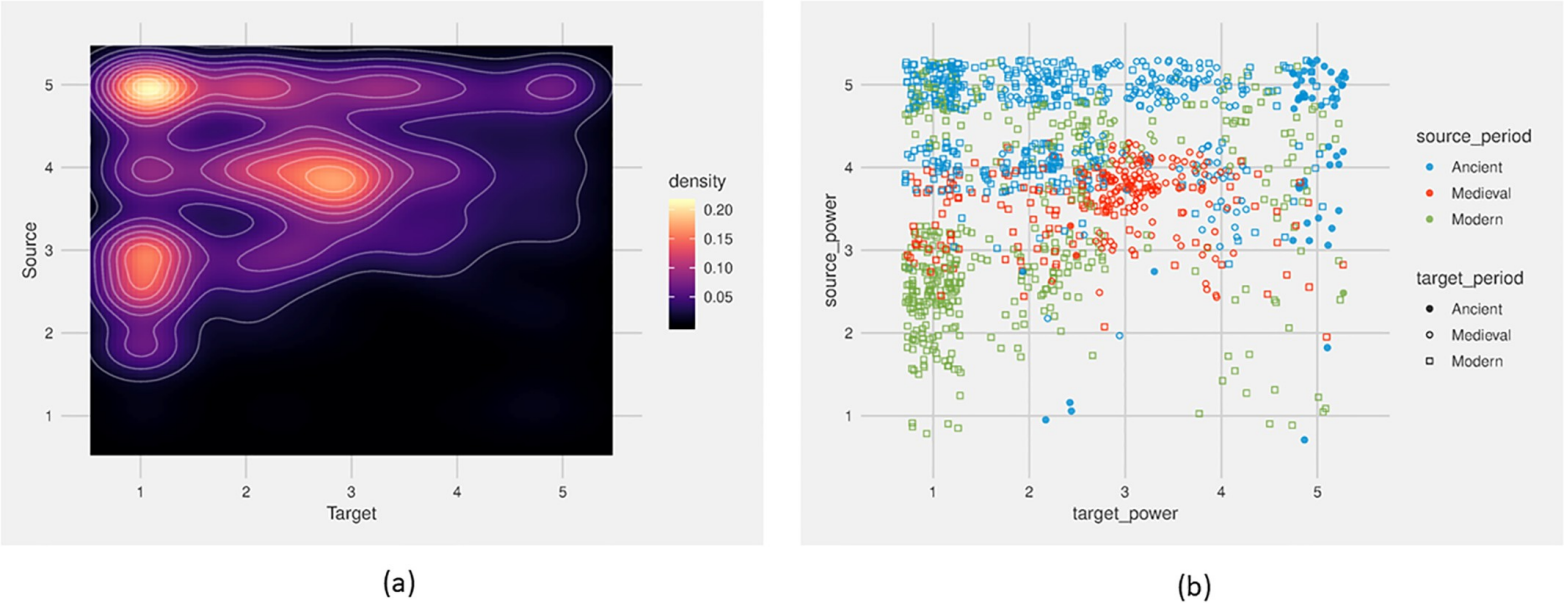
Heatmaps of source and target languages of loan events in relation to Language Power Index. (a) Density heatmap indicating the frequency of languages as source (y) and target (x) language in loan events, by their ranking in the Language Power Index rank. Lighter areas indicate higher density of occurrence of loans. (b) Heatmap of Fig 12A (frequency of languages as source (y) and target (x) language in loan events, by their ranking in the Language Power Index rank), with source and target languages defined by their time period: ancient (- 500 ACE), medieval (500–1500 ACE), and modern (1500–2000 ACE).

To measure the correlation between source language and target language by LPI rank, we create a density plot measuring the co-occurrence of source and target language in all our loan events by the LPI rank of the respective languages (Fig 12A). We see a clear tendency: languages with a higher LPI rank are more “hot” in terms of both source and target language, whereas languages with a lower LPI rank are completely “cold” as source language. The diagram has three density hotspots. The first and most evident hotspot is a loan from a highly prestigious language to a weak one (5 → 1). This is apparently our most frequent type of loan event. The second hotspot is from a medium language to a weak one (3 → 1). Both of these hotspots are indicative of the importance of language power in directionality of borrowing. However, they are also indicative of the skewed distribution of power in the data. The languages with the lowest LPI rank make up almost half of the languages in our corpus, whereas the most prestigious languages make up the smallest group. Our third hotspot, representing loans from a slightly more powerful language to a slightly less powerful language (4 → 3) is very interesting. To figure out the patterns of borrowing, we have defined the borrowing events by time period of the source and target languages, and plotted the result in a heat map (Fig 12B). We notice that modern loan events are found over the entire spectrum of events, most frequently in the hotspots most powerful to weakest (5 → 1), but also medium powerful to weak (3 → 1). Ancient to modern loans, represented e.g., by Latin loans in modern languages, occur frequently in the powerful to weak group (5 → 1), whereas ancient to ancient loans are frequent in the powerful to powerful cluster (5 → 5, 4, 3). The medieval loans are concentrated in the hotspot of medium to medium power loans (4 → 3).

## Discussion

The current paper aims to understand the mechanisms behind linguistic borrowing. First, we reflect on what kinds on conclusions can be drawn from a relatively limited study such as ours. We investigate 104 concepts of high age and cultural salience, which have been in everyday use in Eurasia *at least* since the Secondary Products Revolution (4000–3000 BCE) during the early Chalcolithic, a period that led to many important innovations, such as traction, milking, and the invention of the wheel and plough [66].

Our model aims to capture core borrowings, i.e., loans that replace a lexeme designating a concept previously existing in the language, and the results are therefore not indicative of expansion of the vocabulary by cultural borrowings [15], which is another important purpose of lexical borrowing. At some point in history, parts of our targeted vocabulary must have entered the lexicon by cultural borrowing, when new words entered the languages together with new, recently invented concepts. The source languages of these loan events are mostly unknown to us.

Furthermore, our targeted concept list mainly encompasses the areas of subsistence, technology, hunting and war (Table 1). These are interesting and important areas from a cultural classification perspective [33, 67], but these domains are not representative of the entire lexicon. The highly diverging results for the lexemes pertaining to the environment, such as terms for wild animals (e.g., bison, wild boar, wolf), in contrast to related domestic animals (e.g., cow, pig, dog), indicates that the borrowability variation in some domains is very complex.

Finally, our study is restricted to a specific macro-area and one continent, and the results are therefore only indicative of general trends. As we have seen, our results diverge from the results of the Loanword Typology Project [4], which targets a global sample, in that our borrowability rates are much *lower* (Fig 7). This can be explained by the fact that our cultural concepts are native to the Eurasian historical subsistence system and environment and are naturally loans in other parts of the world. Our cultural explanation model uses the farming settlement as a basis for increased borrowability (Fig 2). The settlement is culturally of high importance to societies which have farming as their main source of subsistence [44], and the importance of centre and periphery for social interaction goes beyond the settlement in these societies [43]. It is thus predictable that this fundamental organizational basis of society impacts language change, including levels of borrowability. It is questionable whether this scenario is transferable to other subsistence systems, such as foragers or hunter-gatherers. However, the significant correlation between the borrowability rates of the global sample LTP and our data indicate that borrowability, also in cultural vocabulary, may be governed by general, “universal” principles.

In linguistic literature, the reasons for borrowing are explained as either *need* or *prestige*. To understand this variability, we use two models of quantification. For the causality need, which we define by a cultural “distance-from-hearth” model (Fig 2), we find that increasing cultural involvement and labour increases the level of borrowability. This correlation is significant. As expected, differing levels of borrowability in the environmental category can be explained by the spatial distribution of natural habitats of some of the animals: for instance, jackals, leopards, lynx, and lions are not native to large parts of our area. Here, we have aimed at a weighed selection, covering the entire area [62]. On the other hand, game animals such as deer, bison, rabbits, hares, and foxes are found in all of our area, and still they show highly variable levels of borrowability.

Moving over to prestige, our results show a predictable and interesting outcome. We create a model for quantifying socio-economic and cultural prestige of languages of various periods and sizes. Using this ranking system, we investigate the directionality of borrowing by looking at loan events between a source language and a target language. We find that all languages are basically equally vulnerable to borrowing, independent of prestige (Fig 10). In principle, English and Latin borrow as much as a small minority language in the Caucasian mountains. This is expected. On the other hand, we find a significantly positive correlation between socio-economic and cultural prestige and occurrence as source language: more prestigious languages are significantly more frequent as source languages (Fig 11). This is expected, but there are some exceptions. Languages that are locally important as source languages, such as Swedish (in Northern Europe), Azerbaijani or Georgian (in the Caucasus), have medium ranks for prestige but are more frequent in our data as source languages than expected according to our model.

Finally, if we consider the occurrence as source and target languages in relation to prestige, we realize that there is a clear connection between power and directionality of borrowing (Fig 12A). The heat map indicates that there is a “hot” and a “cold” side when it comes to borrowing. Weak languages (from 1–2) are ice-cold as source languages. More prestigious languages (3–5) occur as source as well as target languages. Most frequently, a less prestigious language borrows from a more prestigious one. However, the results show some interesting deviations, most importantly the three hot-spots of loan directionality, very powerful to very weak (5 → 1), medium powerful to weak (3 → 1), and medium strong to medium (4 → 3). To try to contextualize this result, we display the result by time period of the source and target languages of loan events (Fig 12B). Most of the loan events coincide in time, but a number of loan events are from languages of previous periods into languages of later periods. We notice that modern loans are found in all types of events. However, the hotspot of medium powerful loan events (4 → 3) is mainly composed by medieval loan events, both as source and as target language (Fig 12B). There are several possible explanations for the patterns. The first and most important is the availability of data. Weak languages from historical periods are typically not preserved, but this goes for ancient as well as medieval languages and cannot account for the difference here. Another possible explanation is that the communication and linguistic exchange during the Middle Ages, which was a formative time for the modern Eurasian languages [68], involved languages which were relatively equal in power, both in the west as well as in the east. The uneven impact of a few powerful languages on a larger number of smaller languages is result of the periods preceding (i.e., 500 BCE– 500 ACE) and following (1500 ACE—now) the Middle Ages. An uneven relationship is clearly present during the ancient period, where a handful of very prestigious languages, such as Classical Greek, Latin, and Sanskrit, dominate the literary record. The dominance of a handful of prestigious languages in the modern period (English, Russian, French, etc.) is the result of the colonization period (16^th^-18^th^ centuries). Another possible explanation is that our ranking of the medieval languages not entirely appropriate (all historical rankings are evaluative rankings), and that the ranking motivations have not been sufficient to capture power relations during this period. In any case, our results reflect the uniqueness of the Eurasian area, with its long history of written records. We would expect a completely different result for other parts of the world, and a study such as ours may not even be possible on some continents.

## Conclusion

We investigate lexical borrowing on an empirical dataset of 115 Eurasian languages from 7 different families, where most languages belong to the Indo-European family. We use a cognacy coded dataset, where loans have been marked, identifying a number of lexical loan events including a source and a target language, which are defined by three time periods: ancient (– 500 ACE), medieval (500–1500 ACE) and modern (1500 ACE–). To promote continuity, and to avoid loans that coin terms for new concepts entering the language, we use a list of lexical concepts within the spheres of subsistence, which have been in daily use at least since the Chalcolithic (4000–3000 BCE). To investigate need and prestige, we organize concepts and languages according to two different ranks. The first rank, the Culture Labour Intensity rank, classifies concepts by their cultural function, defined by the qualities and functions Ground<Figure, Farming<Industry, Small-scale<Large-scale, Indoor<Out-door, referring to increased labour and cultural involvement. The second rank, the Language Power Index rank, defines the socio-economic and cultural prestige of languages by means of a quantitative and evaluative system including population size, economic impact, level of colonization, literary power, and level of extension in prehistory. We analyse the rates of borrowing by concept, semantic domain, language, family, and time period. We compare our result to a previous study of borrowability using a non-genetic, global sample, concluding that the borrowing scores in our data are on average lower. We explain this by the fact that many of our concepts are native to Eurasian languages, whereas they are borrowed from colonizing languages outside of the Eurasian continent. However, the borrowability levels of concepts are significantly correlated between our data and the global sample, which indicates that some patterns of borrowability are general and independent of local cultural adaptation. In two separate studies we test the borrowability rates of concepts and languages in relation to our rankings. We find that the level of borrowability of concepts increases accordingly with an increasing labour intensity, where concepts pertaining to the household and small-scale farming have the lowest scores, concepts pertaining to large-scale farming have higher scores, and concepts pertaining to industry and technology the highest scores. Concepts pertaining to the environment vary depending on their property of ability of relocating and their involvement in culture. As for linguistic prestige and loan directionality, we find that socio-economic and cultural prestige predicts the directionality of borrowing, but it is not evenly distributed over time, which may be an artefact of the availability of data for historical languages. Alternatively, the result is indicative of more uneven power relations between loan-givers and loan-takers during the ancient and modern periods, and more balanced power relations during the middle ages. In general, need, defined as cultural labour and involvement, and prestige, defined as socio-economic and cultural power, are strongly correlated with lexical borrowability.

## Supporting information

S1 Appendix Languages and language metadata. (XLSX)

S2 Appendix Lexical data, including loan coding. (XLSX)

S3 Appendix Data for the Culture Labour Intensity (CLI) rank. (XLSX)

S4 Appendix Data for the Language Power Index (LPI) rank. (XLSX)

S5 Appendix Borrowability statistics for languages and concepts. (XLSX)

S1 File Literary sources for cognacy and loan judgements in the data S2 Appendix. (PDF)

S2 File Validation test of factors of the Culture Labour Intensity (CLI) rank (S3 Appendix). (PDF)
