# Supplementary material for: The causality of borrowing: Lexical loans in Eurasian languages
Source: PLoS One. 2019 Oct 30;14(10):e0223588. doi: 10.1371/journal.pone.0223588 (PMC6821065; doi:10.1371/journal.pone.0223588)
Supplement: S1 File — (PDF) [file pone.0223588.s006.pdf]

Appendix 6. Literary sources to cognacy and loan coding

Abaev, Vasilij I. 1970. Russko-osetinskij slovar' [Russian-Ossetian dictionary]. Moskva: Sovetskaja Enciklopedija.

Achaṛean, Hrach'eay & Nersisi͡an, Mkrtich Gegamovich. 1979. Hayerēn armatakan baṙaran [Dictionary of Armenian Root Words], 2nd edn. Yerevan: Yerevan State University.

Adams, Douglas Quentin. 2013. Etymological Dictionary of Tocharian B. Amsterdam - Atlanta: Rodopi.

Alcover, Antoni Maria & Francesco de Borja Moll. 1930-1962. Diccionari català-valencià-balear : inventari lexicogràfic i etimològic de la llengua catalana en totes les seves formes literàries i dialectals. Palma de Mallorca: Editorial Moll.

Alexander, Ronelle & Ellen Elias-Bursać. 2010. Bosnian, Croatian, Serbian, a Textbook, 2nd edn. Madison: The University of Wisconsin Press.

Aliyev, Elnur. 2017. Fieldwork data on Caucasian languages.

Aliyev, Elnur. to appear. Trilingual (Kryz-Azerbaijani-English) Electronic Dictionary.

Allén, Sture & Åsa Abelin. 1986. Svensk ordbok: ordens betydelse och användning: mer än 100.000 ord och fraser [Swedish dictionary: the meaning and use of words: more than 100.000 words and phrases]. Solna: Esselte studium.

Analyse et traitement informatique de la langue française (ATILF). 2015. DMF = Dictionnaire du Moyen Français. Nancy: ATILF - CNRS & Université de Lorraine. http://www.atilf.fr/dmf

Analyse et traitement informatique de la langue française (ATILF). 2014-2018 Dictionnaire Électronique de Chrétien de Troyes. Nancy: ATILF. http://www.atilf.fr/dect/

Ardoteli 2010. Nodar Ardot'eli. Bežit'uri ena (gramat'ik'uli analizi, t'ekst'ebi, leksik'oni) [Bezhta language (grammatical analysis, texts and dictionary)]. Tbilisi 2010. 209 gv.

Ardoteli 2017. Nodar Ardot'eli. Bežit'uri-kartuli leksik'oni (lingvist'uri k'oment'arebit, indeksita da gramat'ik'uli nark'vevebi) [Bezhta-Georgian dictionary (with linguistic comments, index and grammatical outline)]. Tbilisi 2017. 340 gv.

Bailey, Matthew (trans., ed.). 2004. Cantar de mio Cid. Austin: University of Texas. http://www.laits.utexas.edu/cid/

Bartholomae, Christian. 1979 [1904-1906]. Altiranisches Wörterbuch: zusammen mit den Nacharbeiten und Vorarbeiten. Berlin - New York: Mouton de Gruyter.

Bədəlov, Hacıbala, Azay Əliyev & İldırım Zeynalzadə. 2014. Azerbaijani-English-Khinaliq Phrasebook. Khinalug.

Beekes, Robert. 1988. A Grammar of Gatha-Avestan. Leiden: Brill.

Beekes, Robert. 2010-. Etymological Dictionary of Greek. Leiden: Brill. http://dictionaries.brillonline.com/greek

Bhaldraithe, Tomás de. 1959. English-Irish dictionary: with terminological additions and corrections. Dublin: An Gúm.

Bibliographisches Institut GmbH. 2013. Duden Die deutsche Rechtschreibung (online). http://www.duden.de/

Blau, Joyce. 1980. Manuel de kurde: dialecte Sorani. Grammaire, textes de lecture, vocabulaire kurde-français et français-kurde. Paris: Klincksieck.

Blažek, Václav. 2009. On the position of Gaulish within Celtic from the point of view of glottochronology. In Benjamin W. Fortson, Götz Keydana, Elisabeth Rieken & Paul Widmer (eds.), Indogermanische Forschungen 114(1), 257-299. Berlin: de Gruyter.

Blažek, Václav. 2010. Glottochronologic Analysis of the Greek Lexicon: Modern, Tsakonian, Old and Mycenaean Greek. In Graeco-Latina Brunensia, 15(1), 17-35.

Blažek, Václav & Irén Hegedűs. 2012. On the position of Nuristani within Indo-Iranian. In Roman Sukac & Ondrej Sefcik (eds.), The Sound of Indo-European 2: Papers on Indo-European phonetics, phonemics and morphophonemics, 40-66. München: LINCOM.

Blažek, Václav. 2013a. Albanian Swadesh Etymology. Unpublished manuscript.

Blažek, Václav. 2013b. Armenian Swadesh Etymology. Unpublished manuscript.

Blažek, Václav. 2013c. Rhaeto-Romance languages: Overview and classification. Unpublished manuscript.

Blochet, Edgar. 2005. Studies in the Pahlavi grammar. Kolkata: The Asiatic Society.

Bomhard, Allan R. 2010-. Reconstructing Proto-Nostratic: Comparative Phonology, Morphology, and Vocabulary. Leiden: Brill. http://dictionaries.brillonline.com/proto-nostratic

Böning, Hermann. 1941. Plattdeutsches Wörterbuch für das Oldenburger Land. Oldenburg: Gerhard Stalling.

Borchling, Conrad, Gerhard Cordes, Agathe Lasch & Dieter Möhn. 1928-. Mittelniederdeutsches Handwörterbuch. Neumünster: Wachholtz Verlag.

Bosworth, Joseph. 1882-1898. An Anglo-Saxon dictionary based on the manuscript collections of Joseph Bosworth. Oxford: Clarendon Press.

Boutkan, Dirk & Sjoerd Michiel Siebinga. 2010-. Old Frisian Etymological Dictionary. Leiden: Brill. http://dictionaries.brillonline.com/frisian

Boyle, John Andrew. 1973. The hare in myth and reality: a review article. In Folklore, 84(4), 313-326.

Bhaldraithe, Tomás de. 1959. English-Irish dictionary: with terminological additions and corrections. Dublin: An Gúm.

Buck, Carl Darling. 1949. A dictionary of selected synonyms in the principal Indo–European languages: a contribution to the history of ideas. Chicago: University of Chicago Press.

Carling, Gerd. 2009. Dictionary and Thesaurus of Tocharian A. Volume 1: A-J. Wiesbaden: Otto Harrassowitz.

Carling, Gerd. 2016-2018. CeDICT = A Comprehensive e-Dictionary of Tocharian (online). Lund: Lund University, The Faculties of Humanities and Theology.

Carreras i Martí, Joan (ed.).1983. Diccionari de la llengua catalana [Dictionary of the Catalan language], 2nd edn. Barcelona: Institut d'estudis catalans. http://dlc.iec.cat/

Cassignac, Arve. 2015. Dictionnaire Francais - Occitan, Occitan - Francais, Occitan de communication. Toulouse: Mobileoccitan. http://www.panoccitan.org/

Charyjarov, B. & C. Altaev (eds.). 1986. Bol’šoj Russko-Turkmenskij Slovar' [The Big Russian-Turkmen Dictionary].

Cheung, Johnny. 2010-. Etymological Dictionary of the Iranian Verb. Leiden: Brill. http://dictionaries.brillonline.com/iranian

Chrelashvili, Kote. 2002. Tsovatushuri ena [Tsova-Tush language]. Tbilisi: Tiblisi University Press.

Chukhua, Merab. 2016. Unpublished draft of cultural vocabulary in the Caucasus.

Cioranescu, Alexandre. 1958-1966. Diccionario etimológico rumano. La Laguna: Universidad de La Laguna.

Cleasby, Richard. 1874. An Icelandic-English dictionary. Oxford: Clarendon Press.

Collins English Dictionary: complete and unabridged, 6th edn. 2003. New York: HarperCollins.

Collins German dictionary, 5th edn. 2004. London: Collins.

Comrie, Bernard & Madzhid Khalilov. 2009. Loanwords in Bezhta, a Nakh-Daghestanian language of the North Caucasus. In Martin Haspelmath & Uri Tadmor, Loanwords in the World’s Languages: A Comparative Handbook, 414-429. Berlin: De Gruyter Mouton.

Comrie, Bernard & Madzhid Khalilov. 2010. Dictionary of languages and dialects of the peoples of the Northern Caucasus: comparison of the basic lexicon. Machačkala: Dagestanskij Naucnyj Centr Ross. Akad. Nauk.

Cornish Language Partnership online dictionary. 2015. http://www.cornishdictionary.org.uk/

Corominas, Joan. 1991-1997. Diccionario crítico etimológico castellano e hispánico. Obra completa. Madrid: Editorial Gredos.

Dal’, Vladimir Ivanovich. 1880-1882. Tolkovyj slovar' živago velikoruskago jazyka [Explanatory Dictionary of the Living Great Russian Language], 2nd edn. Moscow: Izdanie knigoprodavcatipografa M. O. Vol'f.

Dehn, Britt-Marie. 1998. Norstedts italienska ordbok, 2nd edn. Stockholm: Norstedt.

Derksen, Rick. 2010-. Etymological Dictionary of the Baltic Inherited Lexicon. Leiden: Brill. http://dictionaries.brillonline.com/baltic

Derksen, Rick. 2010-. Etymological Dictionary of the Slavic Inherited Lexicon. Leiden: Brill. http://dictionaries.brillonline.com/slavic

Desheriev, Julius. 1953. Batsbijskij jazik [Batsbi language]. Moscow: Izdatel'stvo Akademii Nauk SSSR.

Det Danske Sprog- og Litteraturselskab. 2004-. Den Danske Ordbog [The Danish dictionary]. Copenhagen: Det Danske Sprog- og Litteraturselskab. http://ordnet.dk/ddo

DEX online: Dicționare ale limbii române. 2004-2015. https://dexonline.ro/

Dicionário Inglês-Português, Português-Inglês. 2001. [English-Portuguese, Portuguese-English dictionary]. Porto: Porto Editora.

Dinneen, Patrick S. 1904. Foclóir Gaedhilge agus Béarla: An Irish-English dictionary. Dublin: M. H. Gill & Son, Ltd.

Ernby, Birgitta. 2008. Norstedts etymologiska ordbok: 20.000 uppslagsord [Norstedt’s etymological dictionary: 20.000 headwords]. Stockholm: Norstedts akademiska förlag.

Ernout, Alfred & Antoine Meillet. 1951. Dictionnaire étymologique de la langue latine: histoire des mots, 3rd edn. Paris: Klincksieck.

Fähnrich, Heinz. 2007. Kartwelisches etymologisches Wörterbuch. Leiden: Brill.

Falileyev, Alexander. 2000. Etymological Glossary of Old Welsh. Tübingen: Max Niemeyer Verlag.

Farren, Robert. 2017. PIE culture words collection for the DiACL database.

Feist, Sigmund. 1939. Vergleichendes Wörterbuch der gotischen Sprache mit Einschluss des krimgotischen und sonstiger zerstreuter Überreste des gotischen, 3rd edn. Leiden: Brill.

Fernandes, Alexander. 2010. Norstedts portugisisk-svenska ordbok, 1st edn. Stockholm: Norstedt.

Fisiak, Jacek (ed.). 1996. Collins słownik polsko-angielski [Collins Polish-English dictionary]. Warszawa: Polska Oficyna Wydawnicza.

Foras na Gaeilge's New English-Irish Dictionary. 2013-2018. http://www.focloir.ie/en/

Føroysk orðabók (online). 2012-2018. Orðabókagrunnurin. http://www.obg.fo/.

Frisk, Hjalmar. 1974-1972. Griechiches Etymologisches Wörterbuch. Heidelberg: C. Winter.

De Fryske akademy. 1944. Lyts frysk Wirdboek. I, Frysk – Nederlânsk. Bolsward/Boalsert: Osinga.

De Fryske akademy. 1952. Lyts frysk Wirdboek. II, Nederlânsk - Frysk. Bolsward/Boalsert: Osinga.

Gajiev Mezhid. 1950. Russko-lezginskij slovar [Russian-Lezgian dictionary]. Machačkala: Publishing house of the Dagestan branch of the Academy of Sciences of the USSR.

Galimberti Jarman, Beatriz, Roy Russell, Carol Styles Carvajal & Jane Horwood (eds.). 2003. The Oxford Spanish dictionary: Spanish-English/English-Spanish, 3rd edn. Oxford: Oxford University Press.

Ganieva, Faida A. 1974. Leksika Kryzskogo jazyka (iskonnaja i zaimstvovannaja leksika) [Vocabulary of the Kryz language (original and borrowed vocabulary)]. Makhachkala.

Ganieva, Faida A. 2015. Otraslevaja leksika xinalugskogo jazyka [Branch vocabulary of the Khinalug language]. Makhachkala.

Gasanova, Marina A. 2017. Govorim po-tabasaranski [We speak Tabasaran]. Makhachkala.

Gioan, Pierre. 1963. Dictionnaire usuel quillet flammarion, par le texte et par l'image. Paris, Quillet-Flammarion.

Girolamo, Costanzo di & Charmaine Lee. 1998. Avviamento alla filologia provenzale. Rome: Carocci.

Glare, P. G. W. 1982. Oxford Latin dictionary. Oxford: Clarendon Press.

Göksel, Aslı. & Celia Kerslake. 2005. Turkish: a comprehensive grammar. London: Routledge.

Griffiths, Bruce & Dafydd Glyn Jones. 1995. Geiriadur yr Academi: The Welsh Academy English-Welsh Dictionary Online. Cardiff: Univ. of Wales Press. http://geiriaduracademi.org/?lang=en

Gukasyan, Voroshil. 1974. Udinsko-Azerbaydzhansko-Russkiy slovar' [Udi-Azerbaijani-Russian dictionary]. Baku.

Guriev, T. A. (ed.). 2004. Osetino-russkij slovar’ [Ossetian-Russian dictionary], 5th edition. Vladikavkas: Alanija.

Güterbock, Hans G., Harry A. Hoffner & Theo P. J. van den Hout (eds.). 1980-. The Hittite Dictionary of the Oriental Institute of the University of Chicago. Chicago: Oriental Institute of the University of Chicago.

Häkkinen, Kaisa. 2013. Nykysuomen etymologinen sanakirja [Etymological dictionary of Contemporary Finnish], 6th edn. Helsinki: WSOY.

Haust, Jean. 1990 [1933]. Dictionnaire liégeois, 2nd edn. Liège: Vaillaint-Carmanne.

Hehn, Viktor. 1976. Cultivated plants and domesticated animals in their migration from Asia to Europe. Amsterdam : John Benjamins.

Hellquist, Elof. 1948. Svensk etymologisk ordbok, 3rd edn. Lund: Gleerup.

Henry, Victor. 1900. Lexique étymologique des termes les plus usuels du breton moderne. Rennes: J. Plihon et L. Hervé.

Holisky, Dee Ann & Rusudan Gagua. 1994. Tsova-Tush (Batsbi). In Rieks Smeets (ed.), The indigenous languages of the Caucasus. Volume 4: The North East Caucasian Languages, part 2, 147-212. Delmar, NY: Caravan Books.

Holthausen, Ferdinand. 1925. Altfriesisches Wörterbuch. Heidelberg: C. Winter.

Holthausen, Ferdinand. 1934. Gotisches etymologisches Wörterbuch: mit Einschluss der Eigennamen und der gotischen Lehnwörter im Romanischen. Heidelberg: C. Winter.

Hornby, Albert Sydney. 2005. Oxford advanced learner's dictionary of current English, 7th edn. Oxford: Oxford University Press.

Houaiss, Antônio & Mauro de Salles Villar. 2001. Dicionário Houaiss da língua portuguesa. Rio de Janeiro: Instituto Antônio Houaiss.

Instituut voor Nederlandse Lexicologie. 2007-2010. De Geïntegreerde Taalbank [The Integrated Language Bank] (online). http://gtb.inl.nl/

Isaev, N. G. 2004. Russko-Caxurskij razgovornik [Russian-Tsakhur phrase book]. Makhachkala: DGU.

ISLEX (online). 2008-2018 Reykjavík: Stofnun Árna Magnússonar í íslenskum fræðum. http://www.islex.hi.is/

Ismailova, Ė. I. 2011. Russko-Rutul'skij Slovar' [Russian-Rutul dictionary]. Makhachkala.

Itkonen, Erkki (ed.). 1992-2000. Suomen sanojen alkuperä: etymologinen sanakirja [The origin of Finnish words: an etymological dictionary]. Helsinki: Suomalaisen kirjallisuuden seura.

Kadagidze, David & Niko Kadagidze. 1984. c’ova-tušur-kartul-rusuli leksik’oni [Tsova-Tush-Georgian-Russian Dictionary]. Tbilisi: Mecniereba.

Kahl, Heinrich & Heinrich Thies. 2002. Plattdeutsches Wörterbuch: plattdeutsch-hochdeutsch; hochdeutsch-plattdeutsch; plattdeutsche Rechtschreibung, 2nd edn. Neumünster: Wachholtz Verlag.

Karasaev, A. T. & A. G. Matsiev. 1978. Russko-Chechenskij slovar' [Russian-Chechen Dictionary]. Moscow: Russkij Jazyk.

Kasten, Lloyd A. & Florian J. Cody. 2001. Tentative dictionary of medieval spanish, 2nd edn. New York: Hispanic Seminary Medieval.

Karulis, Konstantīns. 2001. Latviešu etimoloģijas vārdnīca [Latvian etymological dictionary]. Rīgā: Avots.

Kent, Roland G. 1953. Old Persian: grammar, texts, lexicon, 2nd edn. New Haven, CT: American Oriental Society.

Key, Mary Ritchie & Bernard Comrie (eds.). 2015. The Intercontinental Dictionary Series. Leipzig: Max Planck Institute for Evolutionary Anthropology. http://ids.clld.org

Klimov, Georgij Andreevič & Madzhid Khalilov. 2003. Slovar' kavkazskix jazykov. Sopostavlenije osnovnoj leksiki [Dictionary of Caucasian Languages. A comparison of the Basic Vocabulary]. Moscow: Vostochnaya Literatura.

Kloekhorst, Alwin. 2010-. Etymological Dictionary of the Hittite Inherited Lexicon. Leiden: Brill. http://dictionaries.brillonline.com/hittite

Kluge, Friedrich. 1960. Etymologisches Wörterbuch der deutschen Sprache. 18. Auflage. Berlin: Walter de Gruyter.

Kotimaisten kielten keskus. 2002-2009. The Álgu database. Sámegielaid etymologaš diehtovuođđu [The Etymological Database of the Saami Languages] (online database). Helsinki: Research Institute for the Languages of Finland. http://kaino.kotus.fi/algu/

Koukkunen, Kalevi. 1990. Nykysuomen sanakirja 8: Vierassanojen etymologinen sanakirja [Dictionary of Contemporary Finnish 8: Etymological dictionary of foreign words], 2nd edn. Porvoo: WSOY:n graafiset laitokset.

Kouyoumdjian, Mesrob G. 1970. A Comprehensive Dictionary Armenian-English. Beirut: Atlas Press.

Kroonen, Guus. 2010-. Etymological Dictionary of Proto-Germanic. Leiden: Brill. http://dictionaries.brillonline.com/proto-germanic

Kriaras, Emmanuel. 2006-2008. Epitome of the Kriaras Dictionary. The concise Dictionary of Medieval Vulgar Greek Literature (1100-1669), Vol. I-XIV (online). http://www.greek-language.gr/greekLang/medieval_greek/kriaras/index.html

Lagadeuc, Jehan. 1867. Le Catholicon de Iehan de Lagadeuc : dictionnaire breton, français et latin publié par R. F. Le Men d'après l'édition de Me Auffret de Quoetqueveran. Lorient: Ed. Corfmat.

Lambton, Ann K. S. 1974. Persian grammar: including key. Cambridge: Cambridge University Press.

Levy, Emil. 1909. Petit dictionnaire provençal-français. Heidelberg: C. Winter.

Lewis, Charlton T. & Charles Short. 1879. A Latin Dictionary. New York: Harper Row.

Lexer, Matthias. 1930. Mittelhochdeutsches Taschenwörterbuch, 19th edn. Leipzig: S. Hirzel.

Lexer, Matthias. 1872-1878. Mittelhochdeutsches Handwörterbuch: Zugleich als Supple. und alphabetischer Index zum Mittelhochdeutschen Wörterbuche von Benecke-Müller-Zarncke. Leipzig: S. Hirzel.

Lexin (online). 1995-2018. Stockholm: Institutet för språk och folkminnen/Kungliga tekniska högskolan. http://lexin.nada.kth.se/lexin/#

Liddell, Henry George & Robert Scott. 1961 [1940]. A Greek-English Lexicon. Oxford: Clarendon Press.

Lübben, August. 1888. Mittelniederdeutsches Handwörterbuch. Norden: Soltau.

Machek, Václav. 1968. Etymologický slovník jazyka českého [Etymological Dictionary of the Czech language]. Prague: Nakladatelství Lidové noviny.

MacKenzie, David Neil. 1971. A concise Pahlavi dictionary. London: Oxford University Press.

Mainzer Akademie der Wissenschaften und der Literatur und der Akademie der Wissenschaften zu Göttingen. 2010. Mittelhochdeutschen Wörterbuch Online (online). http://www.mhdwb-online.de/index.html

Mallory, James Patrick & Douglas Quentin Adams 2006. The Oxford introduction to Proto-Indo-European and The Proto-Indo-European world. Oxford: Oxford University Press.

Martirosyan, Hrach K. 2010-. Etymological Dictionary of the Armenian Inherited Lexicon. Leiden: Brill. http://dictionaries.brillonline.com/armenian

Matasović, Ranko. 2010-. Etymological Dictionary of Proto-Celtic. Leiden: Brill. http://dictionaries.brillonline.com/proto-celtic

Mayhew, Anthony Lawson & Walter William Skeat. 1888. A concise dictionary of Middle English from A.D. 1150 to 1580. Oxford: Clarendon Press.

Mayrhofer, Manfred. 1986-. Etymologisches Wörterbuch des Altindoarischen. Heidelberg: C. Winter.

Mazanaev, Shaban. 2012. Russko-Agul'skij slovar' [Russian-Agul dictionary]. Makhachkala: Izdatel'stvo DGU.

Mejlanova, Unajzat Azizovna. 1984. Budukhsko-russkiy slovar' [Budukh-Russian Dictionary]. Moscow: Nauka.

Melchert, H. Craig. 2010-. Cuneiform Luvian Lexicon. Leiden: Brill. http://dictionaries.brillonline.com/luvian

Metsmägi, Iris, Meeli Sedrik & Sven-Erik Soosaar. 2012. Eesti etümoloogiasõnaraamat [Estonian Etymological Dictionary]. Tallinn: Eesti Keele Sihtasutus. http://www.eki.ee/dict/ety/

Meyer-Lübke, Wilhelm. 1935. Romanisches Etymologisches Wörterbuch, 3rd edn. Heidelberg: C. Winter.

Mistral, Frédéric. 1879-1886. Lou tresor dóu felibrige, ou, Dictionnaire provençal-français: embrassant les divers dialectes de la langue d'oc moderne. Aix-en-Provence: Remondet-Aubin.

Mobili, Robert. 2010. Udinsko-Azerbaydzhansko-Russkiy slovar' [Udi-Azerbaijani-Russian dictionary]. Baku.

Monier-Williams, Monier. 1899. A Sanskrit-English Dictionary: Etymologically and Philologically Arranged with Special Reference to Cognate Indo-European languages, revised by Ernst Leumann, Carl Cappeller et al. Oxford: Clarendon Press.

Nascente, Antenor. 1932. Dicionário etimológico da língua portuguesa [Etymological dictionary of the Portuguese language]. Rio de Janeiro: Francisco Alves.

Nocentini, Alberto. 2010. L'etimologico: vocabolario della lingua italiana, 1st edn. Milano: Le Monnier.

Novog Libera i Srca. 2015. Hrvatski jezični portal [online]. http://www.srce.unizg.hr/arhiva_weba/20141029/www.srce.unizg.hr/gradani/hrvatski-jezicni-portal/index.html

Ó Dónaill, Niall & Bhaldraithe, Tomás de (eds.) 1977. Foclóir Gaeilge-Béarla. Baile Átha Cliath: An Rún.

Olivier, Philipe. 2009. Dictionnaire d'ancien occitan auvergnat: Mauriacois et Sanflorain (1340-1540). Tübingen: DeGruyter.

Orbeliani, Sulkhan-Saba. 1991. Georgian Dictionary, vol. I. Tbilisi

Orbeliani, Sulkhan-Saba. 1993. Georgian Dictionary, vol. II. Tbilisi

Orel, Vladimir E. 1998. Albanian etymological dictionary. Leiden: Brill.

Orucov, Ə. (ed.) 2006. Azərbaycan dilinin izahlı lüğəti. Dörd cilddə. (Tərtibçilər: Əliheydər Orucov, Bəhruz Abdullayev, Nərgiz Rəhimzadə.) Bakı: Şərq-Qərb.

Oxnard, Linda (ed.). 2012. DACCO; The Open Source Catalan-English and English-Catalan Dictionary. www.catalandictionary.org

Pinault, Georges. 1997. Terminologie du petit bétail en Tokharien. In Studia Etymologica Cracovensia 2, 175-218. Krákow: Księgarnia Akademicka.

Pinault, Georges. 2004. Core of Tocharian Etymology (outside pronouns and numerals). Handout, Tocharian course, Freie Universität, Berlin.

Pokorny, Julius. 1959. Indogermanisches etymologisches Wörterbuch. Bern- München: Francke Verlag.

The Princeton Dante Project. 1997-1999. Princeton: Princeton University. http://etcweb.princeton.edu/dante/index.html

Puhvel, Jaan. 1984-2004. Hittite Etymological Dictionary, Vol. 1-6. Berlin - New York: Mouton de Gruyter.

Qarīb, Badr al-Zamān. 1995. Sogdian dictionary: Sogdian-Persian-English. Tehran: Farhangan Publications.

Quin, Ernest Gordon (ed.). 1913-1976. Dictionary of the Irish Language Based Mainly on Old and Middle Irish Materials. Dublin: Royal Irish Academy. http://edil.qub.ac.uk/dictionary/search.php

Real Academia Espanhola. 2014-2018. Diccionario de la lengua española. http://dle.rae.es/?w=diccionario

Rédei, Károly. 1986-1991. Uralisches etymologisches Wörterbuch. Wiesbaden: Harrassowitz.

Rey, Alain & Danièle Morvan. 2005. Le dictionnaire culturel en langue française. Paris: Dictionnaires Le Robert.

Richthofen, Karl. 1961 [1840]. Altfriesisches Wörterbuch. Aalen: Scientia.

Ringe, Don, Tandy Warnow & Ann Taylor. 2002. Indo-European and computational cladistics. In Transactions of the Philological Society, 100(1), 59-129.

Rix, Helmut, Martin Kümmel, Thomas Zehnder, Reiner Lipp & Brigitte Schirmer (eds.). 2001. LIV - Lexikon der Indogermanischen Verben. Die Wurzeln und Ihre Primärstammbildungen. Wiesbaden: Ludwig Reichert.

Rizgar, Baran. 1996. Learn Kurdish: a multi-level course in Kurmanji. London: Bay Foreign Language Books.

Sabhal Mòr Ostaig. 1993. An Stòr-dàta Briathrachais Gàidhlig [The Gaelic Terminology Database]. Sleat, Isle of Skye: Clò Ostaig. http://www2.smo.uhi.ac.uk/gaidhlig/faclair/sbg/lorg.php

Sadeniemi, Matti (ed.). 1979. Nykysuomen sanakirja 4: Vierasperäiset sanat [Dictionary of Contemporary Finnish 4: Words of foreign origin], 5th edn. Porvoo: WSOY:n graafiset laitokset.

Sanikidze, Latavra. 2010. Batsburi (Tsova-tushuri ena) [Batsbi (Tsova-Tush language)]. Tbilisi. (Unpublished work by Sanikidze within the project "Mountain Caucasian languages in Georgia" by the Chikobava institute of linguistics.)

Schützeichel, Rudolf. 1995. Althochdeutsches Wörterbuch, 5th edn. Tübingen: Max Niemeyer Verlag.

Serebriakov, S. B. 1962. Old Georgian – Russian Dictionary. Tbilisi: Publishing House of SSSR Academy of Science.

Simpson, John Andrew & Eva S. Weiner. 1989. Oxford English dictionary. Oxford: Clarendon Press.

Söderwall, Knut Fredrik. 1995 [1887]. Ordbok öfver svenska medeltids-språket. Stockholm: Svenska fornskriftsällskapet.

Spano, Giovanni. 1987 [1851]. Vocabolario sardo-italiano e italiano-sardo: coll'aggiunta dei proverbi sardi. Bologna: Forni.

Starostin, G. (2008) Tower of Babel: An etymological database project. (Available online at: http://starling.rinet.ru). Moscow.

Starostin, S.A., Dybo, A. & Mudrak, O. (2003). Handbuch der Orientalistik. Abt. 8, Zentralasien = Central Asia. Bd 8, Etymological dictionary of the Altaic languages. Leiden: Brill.

State, Nicolae & Cornelia State. 1990. Svensk-rumänsk ordbok = Dicţionar suedez-român [Swedish-Romanian dictionary]. Saltsjöbaden: States förlag.

Steensland, Lars. 2010. Material till en älvdalsk ordbok: svenska-älvdalska, älvdalska-svenska [Material for an Elfdalian dictionary: Swedish-Elfdalian, Elfdalian-Swedish]. Älvdalen: Ulum dalska.

Talibov, Gajiev. 2004. Lezginsko-russkij slovar’ [Lezgian-Russian dictionary]. Makhachkala.

Tandilava, Ali. 2013. Laz dictionary. Tbilisi: Arnold Chikobava Institute of Linguistics.

Tanrikulu, Vildan, Mustafa Aydogan & Mahmut Lewendï. 2006. Svensk-Kurdiskt lexikon (Nordkurdiska), Ferhenga Swêdi-Kurdî (Kurmanci) [Swedish-Kurdish dictionary (Northern Kurdish)]. Stockholm: Myndigheten för skolutveckling.

Ternes, Elmar (ed.). 2011. Brythonic Celtic – Britannisches Keltisch: From Medieval British to Modern Breton. Bremen: Hempen Verlag.

Tobler, Adolf & Erhard Lommatzsch. 1925-. Altfranzösisches Wörterbuch: Adolf Toblers nachgelassene Materialien bearbeitet und mit Unterstützung des Preussischen Akademie der Wissenschaften herausgegeben von Erhard Lommatzsch. Wiesbaden: Deutsche Akademie der Wissenschaften.

Tolman, Herbert Cushing. 1908. Ancient Persian lexicon and the texts of the Achaemenidan inscriptions transliterated and translated with special reference to their recent re-examination. New York: American Book Company.

Trésors de la langue francaise informatisées. 2012-2018. Centre national de ressources techniques et lexiques. http://www.cnrtl.fr/definition/

Turner, Ralph L. 1973 [1966]. A comparative dictionary of the Indo-aryan languages, 2nd edn. London: Oxford University Press.

Universitetet i Oslo in collaboration with Språkrådet. 2010. Bokmålsordboka & Nynorskordboka [Bokmål dictionary & Nynorsk dictionary] (online). Oslo: University of Oslo & Språkrådet. http://www.nob-ordbok.uio.no/perl/ordbok.cgi?

Universitetet i Oslo in collaboration with Språkrådet. 2010. Nynorskordboka [Nynorsk dictionary] (online). Oslo: University of Oslo & Språkrådet. http://www.nob-ordbok.uio.no/perl/ordbok.cgi?

University of Wales. 2002. Geiriadur Prifysgol Cymru: A Dictionary of the Welsh Language. http://welsh-dictionary.ac.uk/gpc/gpc.html

Vaan, Michiel de. 2010-. Etymological Dictionary of Latin and the other Italic Languages. Leiden: Brill. http://dictionaries.brillonline.com/latin

Verdam, Jacob. 1949. Middelnederlandsch handwoordenboek [Middle Dutch hand dictionary]. The Hague: Nijhoff.

Vilborg, Ebbe. 2009. Norstedts svensk-latinska ordbok: 28.000 ord och fraser [Norstedt's Swedish-Latin dictionary: 28.000 words and phrases]. Stockholm: Norstedts akademiska förlag.

Vries, Jan de. 1971. Nederlands etymologisch woordenboek. Leiden: Brill.

Vries, Jan de. 1977. Altnordisches etymologisches Wörterbuch, 3rd edn. Leiden: Brill.

Walde, Alois. 1910. Lateinische etymologisches Wörterbuch, 2nd edn. Heidelberg: C. Winter.

Watkins, Calvert. 2000. The American heritage dictionary of Indo-European roots, 2nd edn. Boston: Houghton Mifflin.

Wright, Joseph. 1902. The English Dialect Dictionary. London: Henry Frowde.

Young, George Vaughan Chichester & Cynthia R. Clewer. 1985. Føroysk-Ensk ordabók: Faroese-English dictionary: with Faroese folk-lore and proverbs and a section by Professor

W. B. Lockwood on Faroese pronunciation. Peel, Isle of Man: Mansk-Svenska Publishing Company.

Zagirov, V. M. 1988. Russko-Tabasaranskij slovar' [Russian-Tabasaran dictionary]. Makhachkala.
