# Supplementary material for: The causality of borrowing: Lexical loans in Eurasian languages
Source: PLoS One. 2019 Oct 30;14(10):e0223588. doi: 10.1371/journal.pone.0223588 (PMC6821065; doi:10.1371/journal.pone.0223588)
Supplement: S2 File — (PDF) [file pone.0223588.s007.pdf]

S7 File. Validation test of factors of the Culture Labour Intensity (CLI) rank

In order to test the stability of each factor of the Culture Labour Intensity rank (see description in “Need: borrowability in relation to the Culture Labour Intensity rank”, S3 Appendix, Table 6), we rebuild the trees of our CART validation test (Fig 9b) by throwing out each of the four component measures. The four resulting trees are given below, throwing out the factors Settlement sphere (a), Subsistence farming type (b), General culture domain (c), and Nature: Ground-figure (d). We notice that General culture domain (c) does not seem to add any information that is not already covered by the other three factors. Based on the accuracy (Table 1), i.e., how many cases that a given tree assigns a correct label, we can suggest the following ranking of order of importance of factors:

1) Settlement sphere
2) Nature: Ground-figure
3) Subsistence farming type

Table 1. Results of the validation test of factors of the Culture Labour Intensity Rank.

Tree	Removed factor	Accuracy	Features left
**(a)**	Settlement sphere	0.73	Nature: Ground-figure
**(b)**	Subsistence farming type	0.92	Settlement sphere + Nature: Ground-figure
**(c)**	General culture domain	1	Settlement sphere + Nature: Ground-figure + Subsistence farming type
**(d)**	Nature: Ground-figure	0.86	Settlement sphere + Subsistence farming type

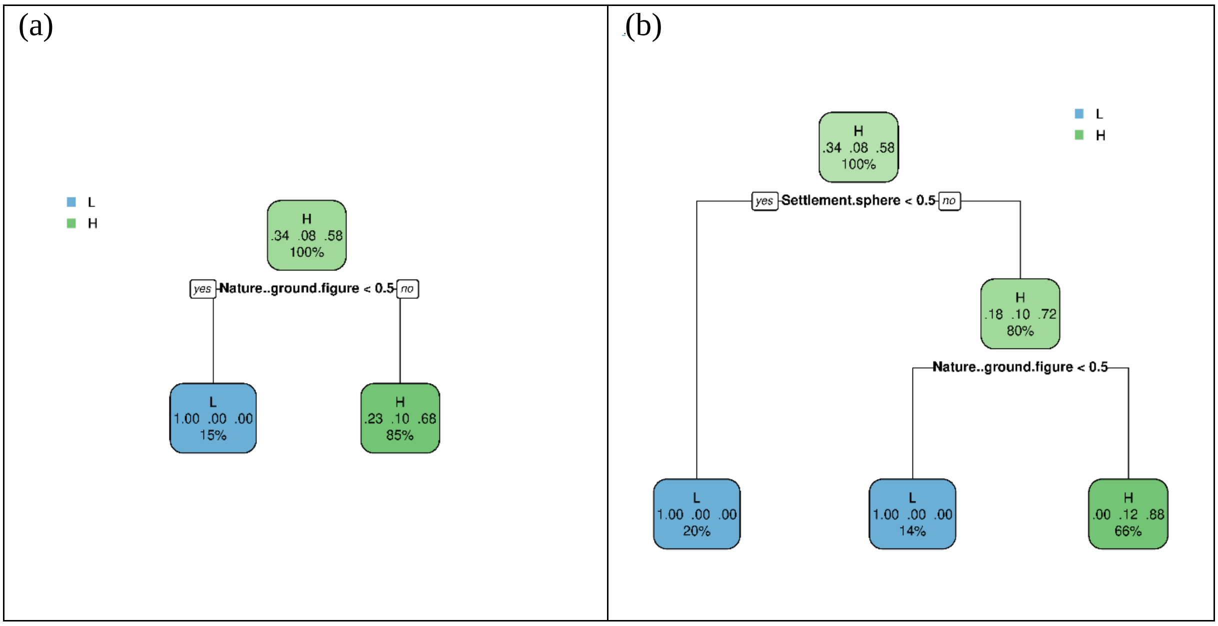

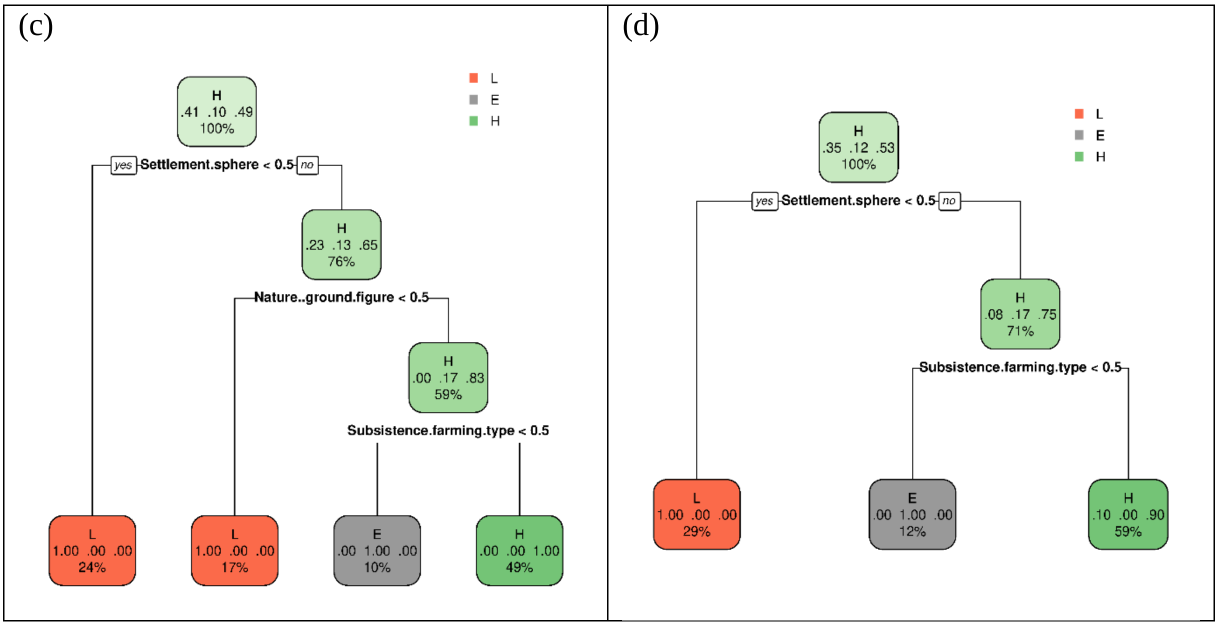
(c)
L
E
H
H
.41 .10 .49
100%
yes
Settlement.sphere < 0.5
no
H
.23 .13 .65
76%
Nature..ground.figure < 0.5
H
.00 .17 .83
59%
Subsistence.farming.type < 0.5
L
1.00 .00 .00
24%
L
1.00 .00 .00
17%
E
.00 1.00 .00
10%
H
.00 .00 1.00
49%
(d)
L
E
H
H
.35 .12 .53
100%
yes
Settlement.sphere < 0.5
no
H
.08 .17 .75
71%
Subsistence.farming.type < 0.5
L
1.00 .00 .00
29%
E
.00 1.00 .00
12%
H
.10 .00 .90
59%
